# Supplementary material for: Atomically precise control of rotational dynamics in charged rare-earth complexes on a metal surface
Source: Nat Commun. 2022 Oct 22;13:6305. doi: 10.1038/s41467-022-33897-3 (PMC9588029; doi:10.1038/s41467-022-33897-3)
Supplement: Supplementary file 1 — Supplementary Information [file 41467_2022_33897_MOESM1_ESM.pdf]

Supplementary Information

Atomically Precise Control of Rotational Dynamics in Charged Rare-Earth Complexes on a Metal Surface

Tolulope Michael Ajayi[1,2], Vijay Singh[3,4], Kyaw Zin Latt[1], Sanjoy Sarkar[2], Xinyue Cheng[5], Sineth Premarathna[1,2], Naveen K. Dandu[3,4], Shaoze Wang[1,2], Fahimeh Movahedifar[5], Sarah Wieghold[1,6], Nozomi Shirato[1], Volker Rose[6], Larry A. Curtiss[3], Anh T. Ngo[3,4], Eric Masson[5,*], & Saw Wai Hla[1,2,*]

[1] Nanoscience & Technology Division, Argonne National laboratory, Lemont, IL 60439, USA.
[2] Nanoscale & Quantum Phenomena Institute, and Department of Physics & Astronomy, Ohio University, Athens, OH 45701, USA.
[3] Materials Science Division, Argonne National laboratory, Lemont, IL 60439, USA.
[4] Chemical Engineering Department, University of Illinois at Chicago, Chicago, IL 60608, USA.
[5] Nanoscale & Quantum Phenomena Institute, and Department of Chemistry and Biochemistry, Ohio University, Athens, OH 45701, USA.
[6] Advanced Photon Source, Argonne National laboratory, Lemont, IL 60439, USA.

*Corresponding authors: Saw Wai Hla (emails: shla@anl.gov, hla@ohio.edu)
Eric Masson (email: masson@ohio.edu)

The submitted manuscript has been created by UChicago Argonne, LLC, Operator of Argonne National Laboratory ("Argonne"). Argonne, a U.S. Department of Energy Office of Science laboratory, is operated under Contract No. DE-AC02-06CH11357. The U.S. Government retains for itself, and others acting on its behalf, a paid-up nonexclusive, irrevocable worldwide license in said article to reproduce, prepare derivative works, distribute copies to the public, and perform publicly and display publicly, by or on behalf of the Government.

Contents

Supplementary Method 1: Characterization of $[Eu(pcam)_3](CF_3SO_3)_3$ and precursors 3

Supplementary Note 1: Distinguishing $[Eu(pcam)_3]^{3+}$ from $[Eu(pcam)_3X]^{2+}$ 13

Supplementary Note 2. Calculated HOMO and LUMO Orbitals of the Complexes 15

Supplementary Note 3. Calculated Density of States 17

Supplementary Note 4. dI/dV Spectroscopy Maps of $[Eu(pcam)_3X_2]^+$ Complex 18

Supplementary Note 5. Calculations of Valence Charges in the Complexes 19

Supplementary Note 6. Controlled Rotation of Charged Rare-Earth Complexes 22

Supplementary Note 7. Evidence of Side Counterion Control Over the Complex 49

Supplementary References 50

<u>Supplementary Method 1</u>. Characterization of [Eu(pcam)$_3$](CF_3SO_3)$_3$ and precursors

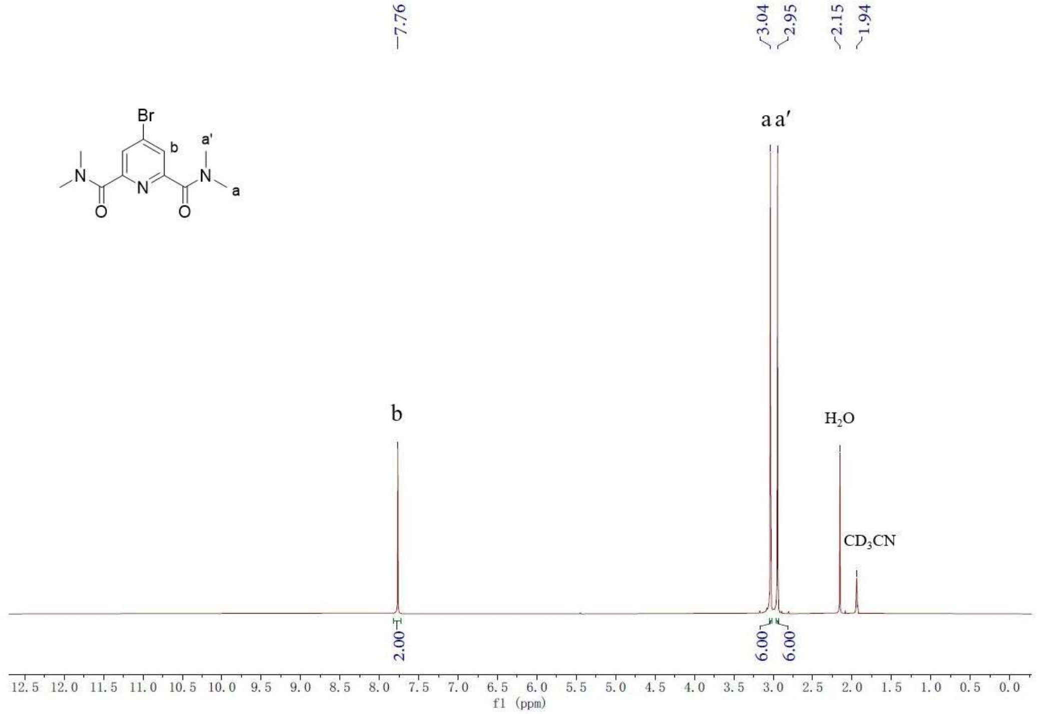

Figure S1. *^{1}H-NMR spectrum of precursor* ***2*** *(500 MHz, CD_3CN, 298 K).*

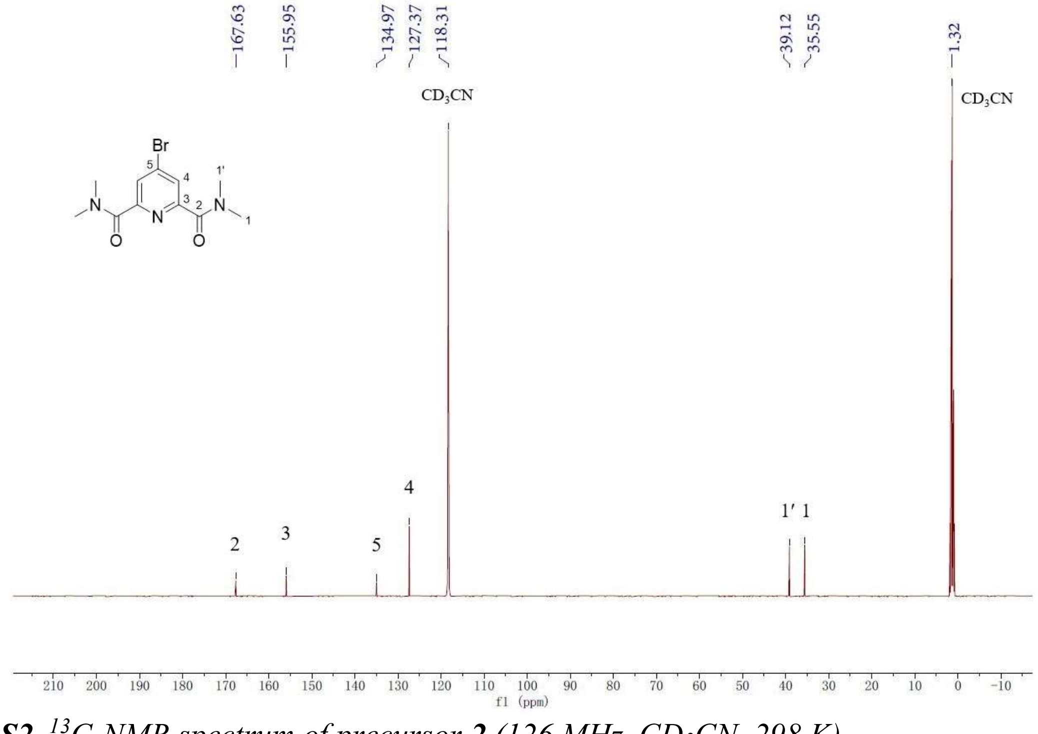

Figure S2. *^{13}C-NMR spectrum of precursor* ***2*** *(126 MHz, CD_3CN, 298 K).*

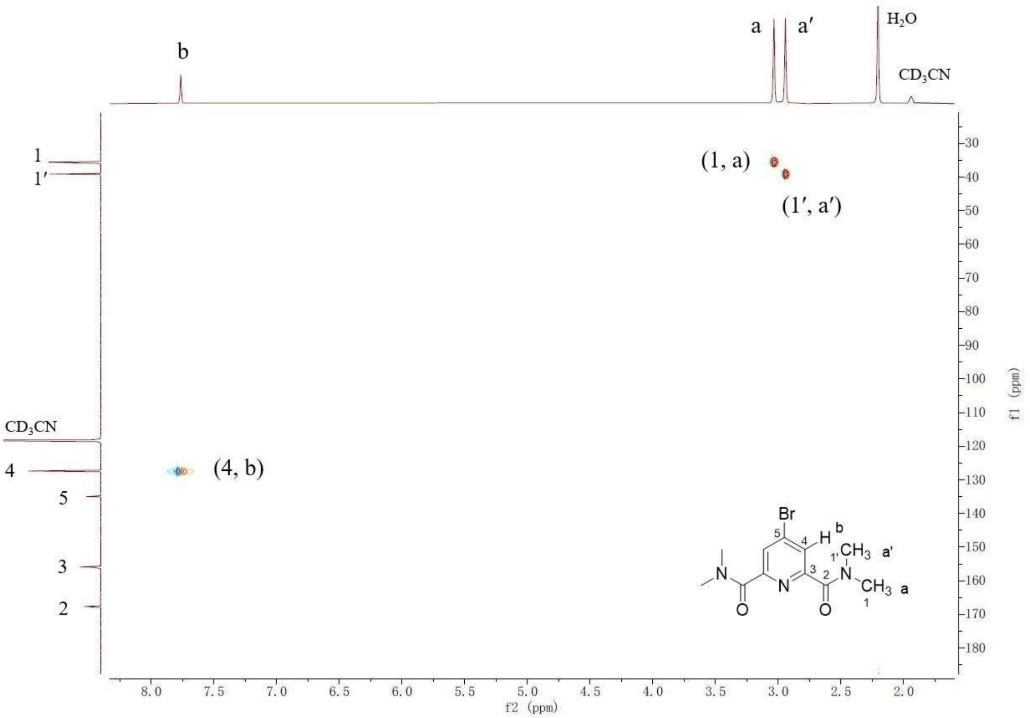

Figure S3. ${}^{1}H$-${}^{13}C$ *HSQC spectrum of precursor* **2** *(300 MHz,* CD_3CN*, 298 K)*

.

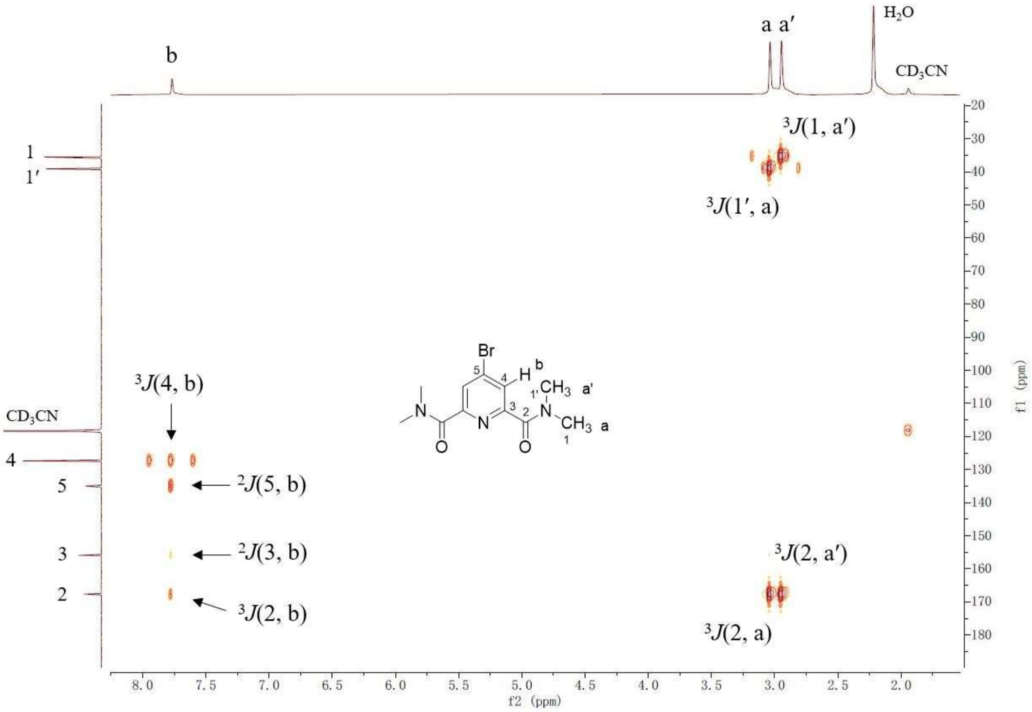

Figure S4. ${}^{1}H$-${}^{13}C$ *HMBC spectrum of precursor* **2** *(500 MHz,* CD_3CN*, 298 K).*

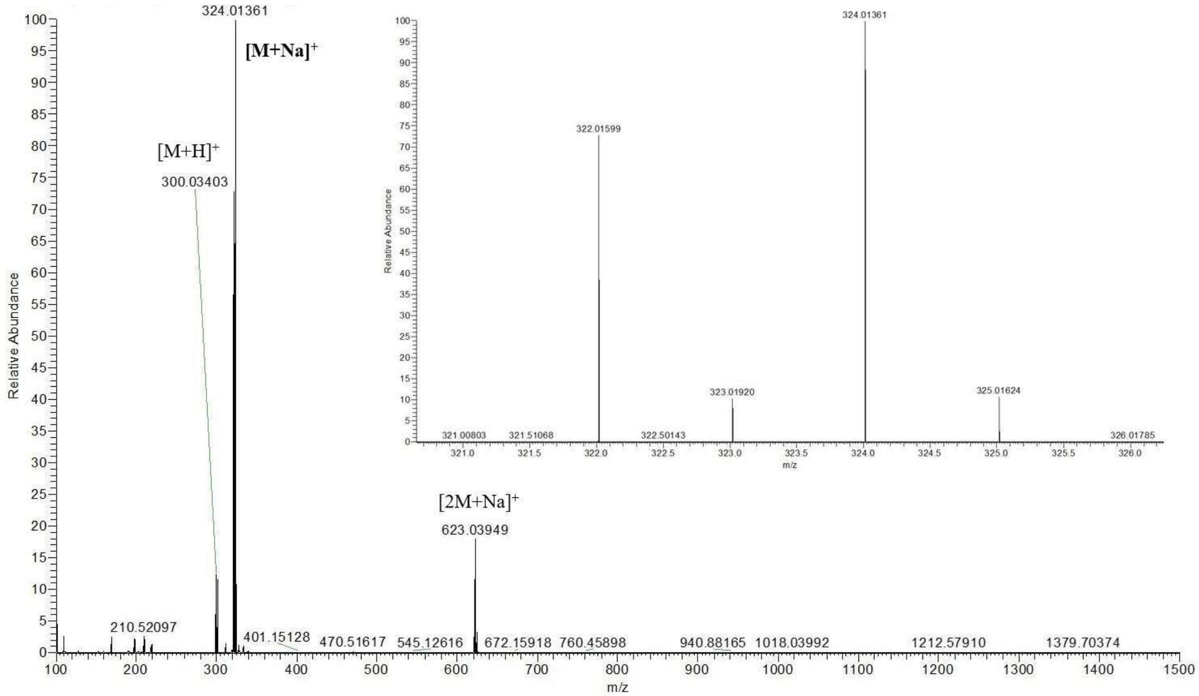

Figure S5. *HR-MS spectrum of precursor **2** in CH_3CN.*

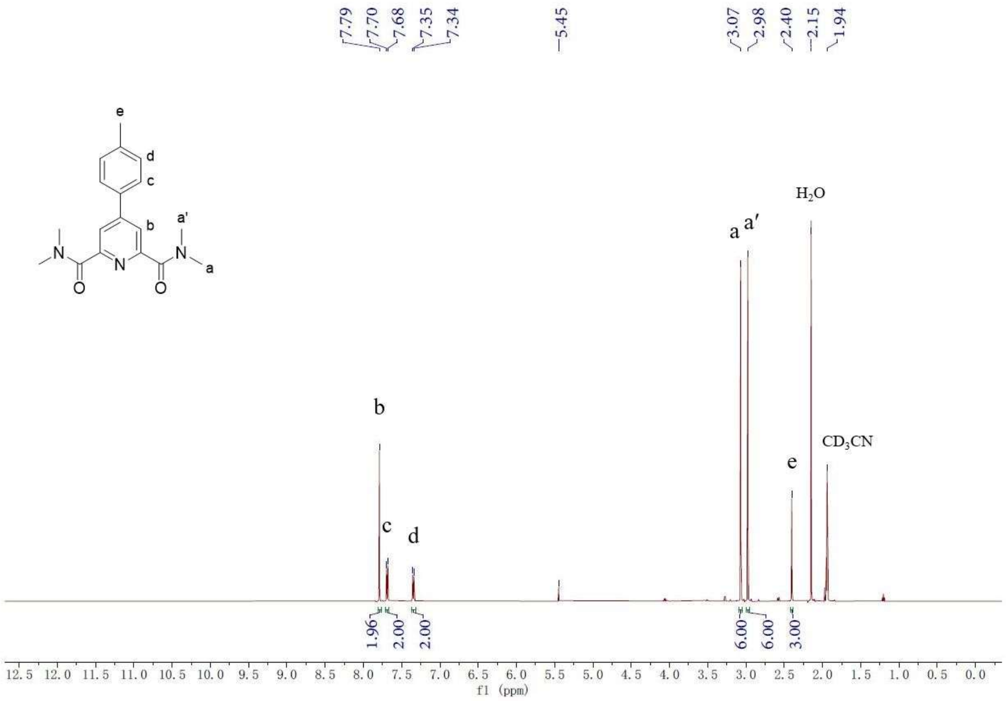

Figure S6. *^{1}H-NMR spectrum of ligand **3** (500 MHz, CD_3CN, 298 K).*

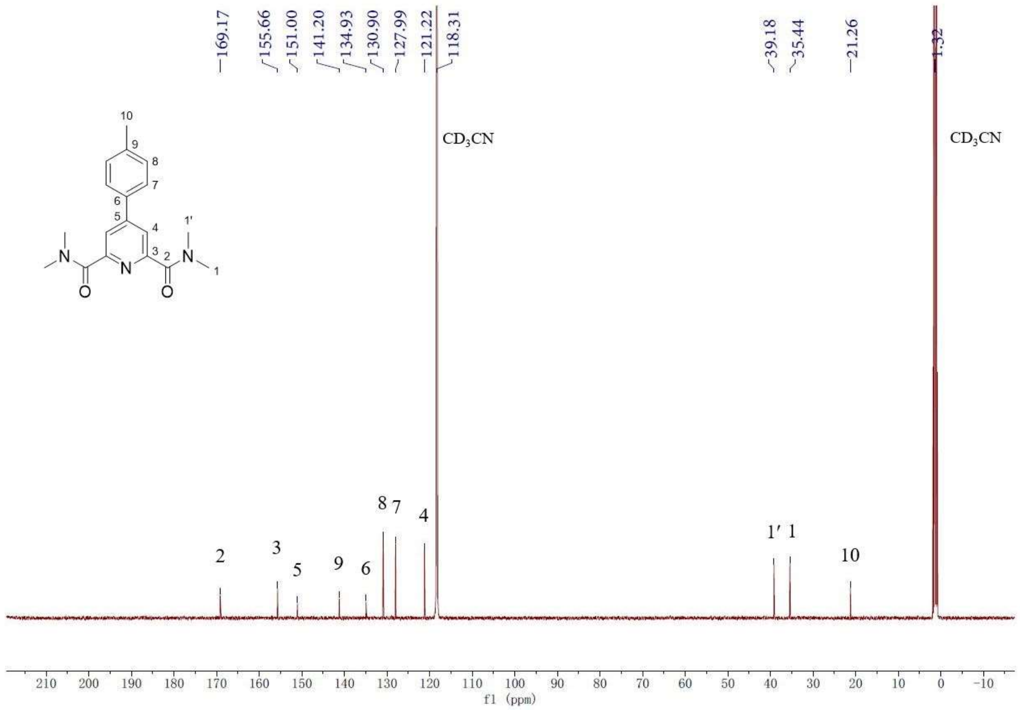

Figure S7. ^{13}C-NMR spectrum of ligand **3** (126 MHz, CD_3CN, 298 K).

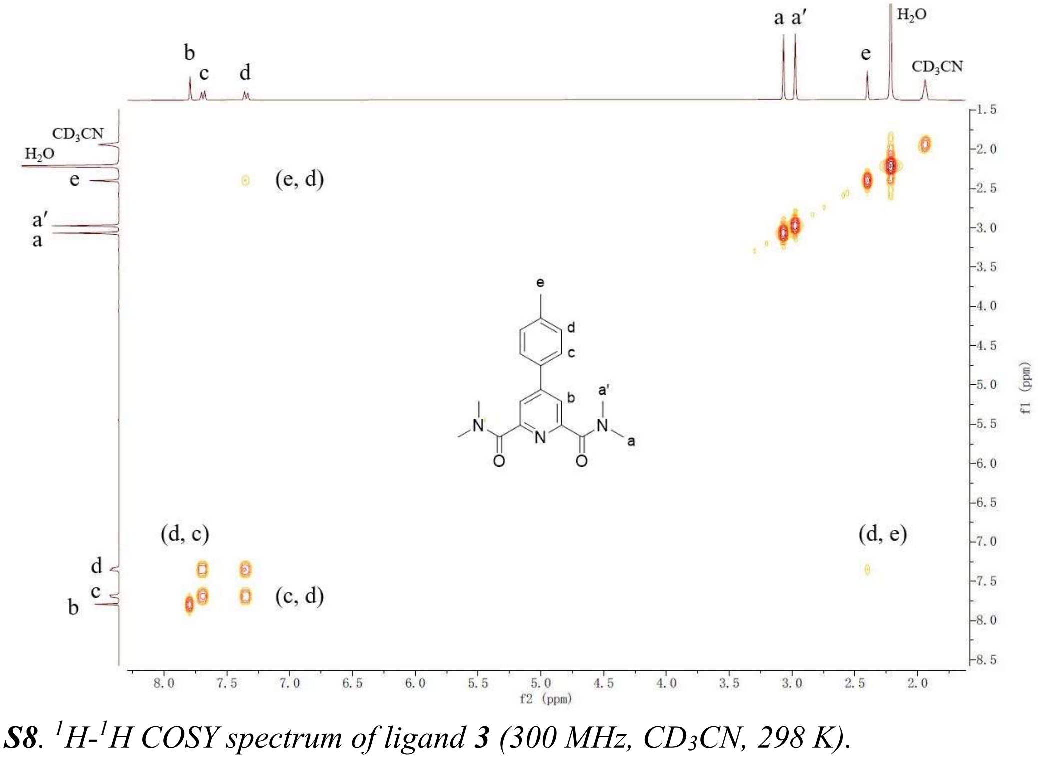

Figure S8. ^{1}H-^{1}H COSY spectrum of ligand **3** (300 MHz, CD_3CN, 298 K).

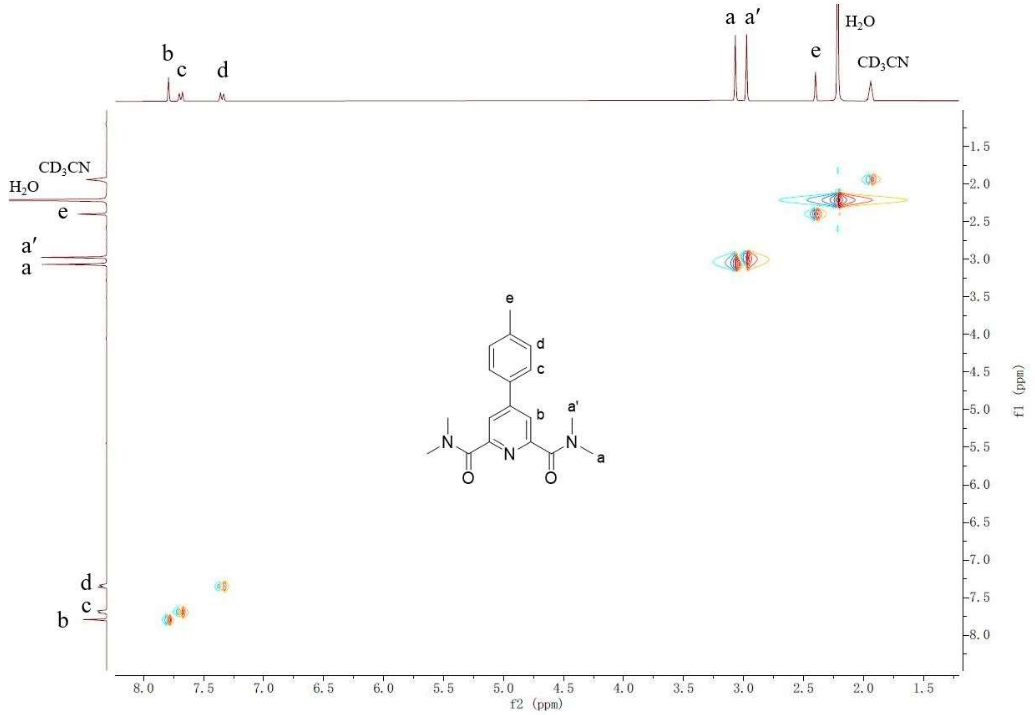

Figure S9. *^{1}H-^{1}H NOESY spectrum of ligand* ***3*** *(300 MHz, CD_3CN, 298 K).*

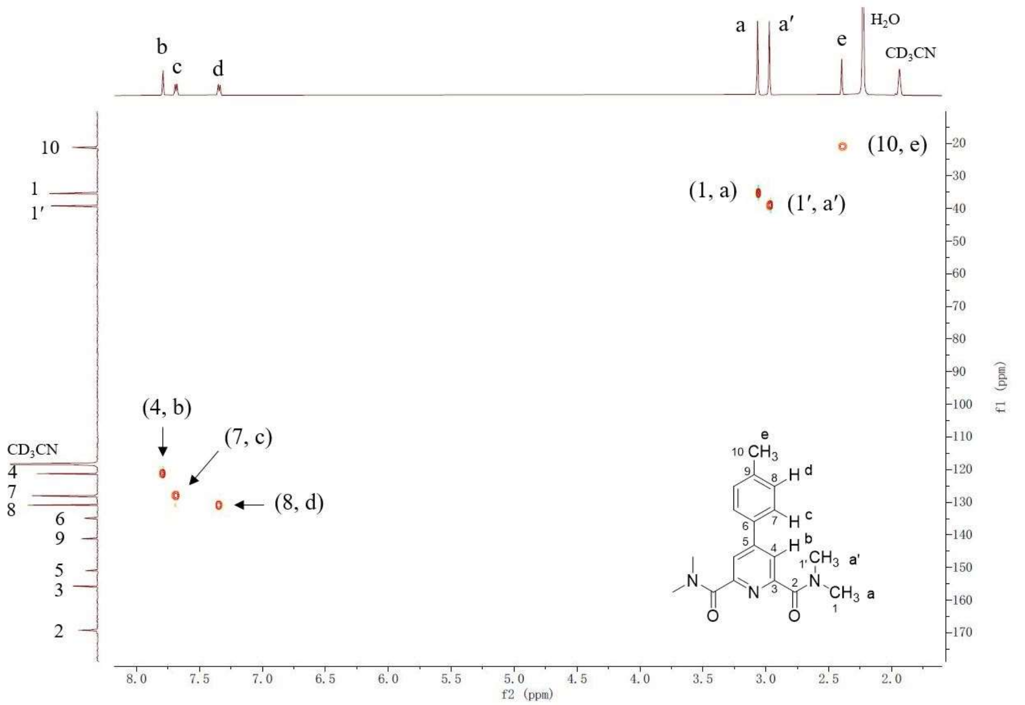

Figure S10. *^{1}H-^{13}C HSQC spectrum of ligand* ***3*** *(500 MHz, CD_3CN, 298 K).*

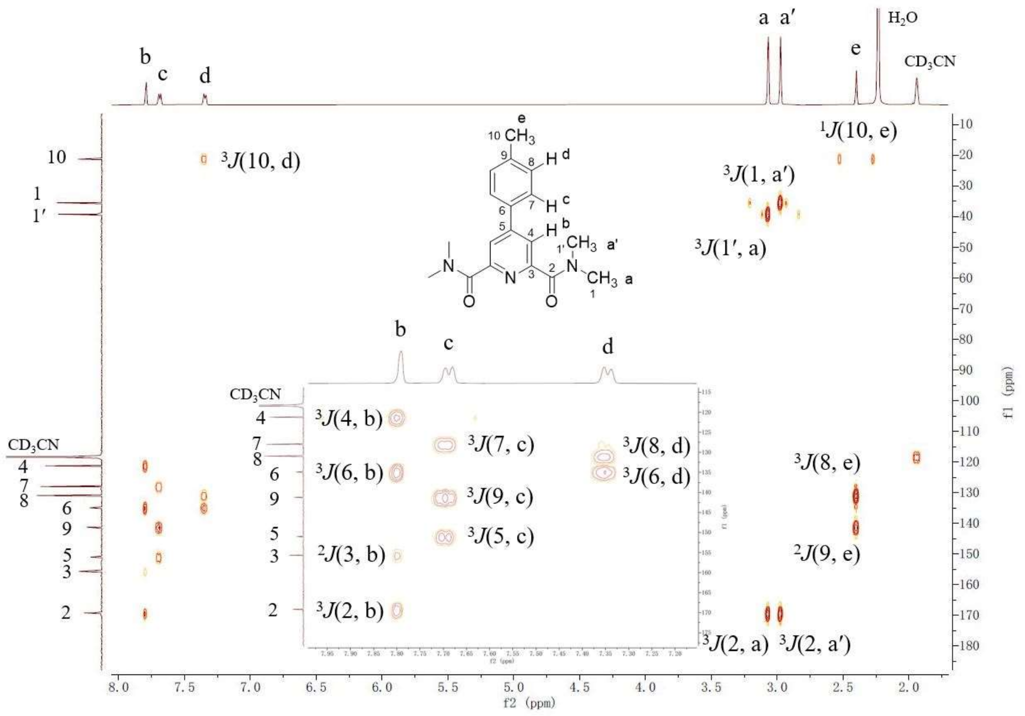

Figure S11. *^1H-^{13}C HMBC spectrum of ligand **3** (500 MHz, CD_3CN, 298 K).*

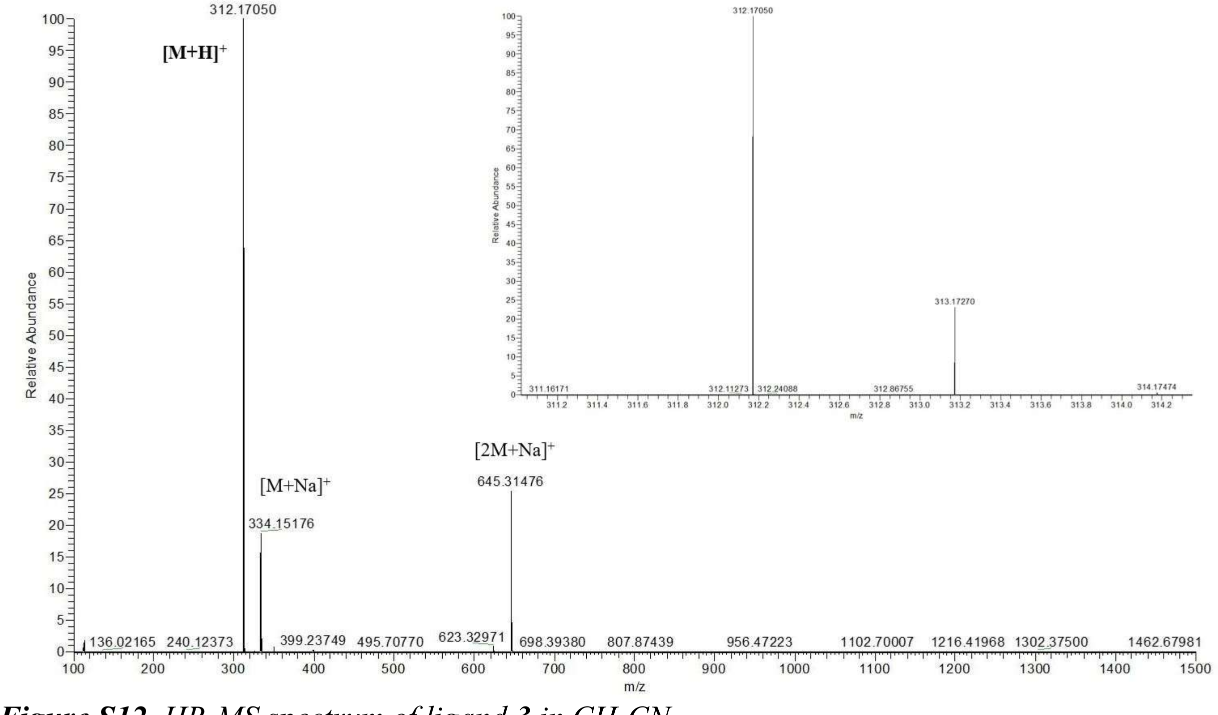

Figure S12. *HR-MS spectrum of ligand **3** in CH_3CN.*

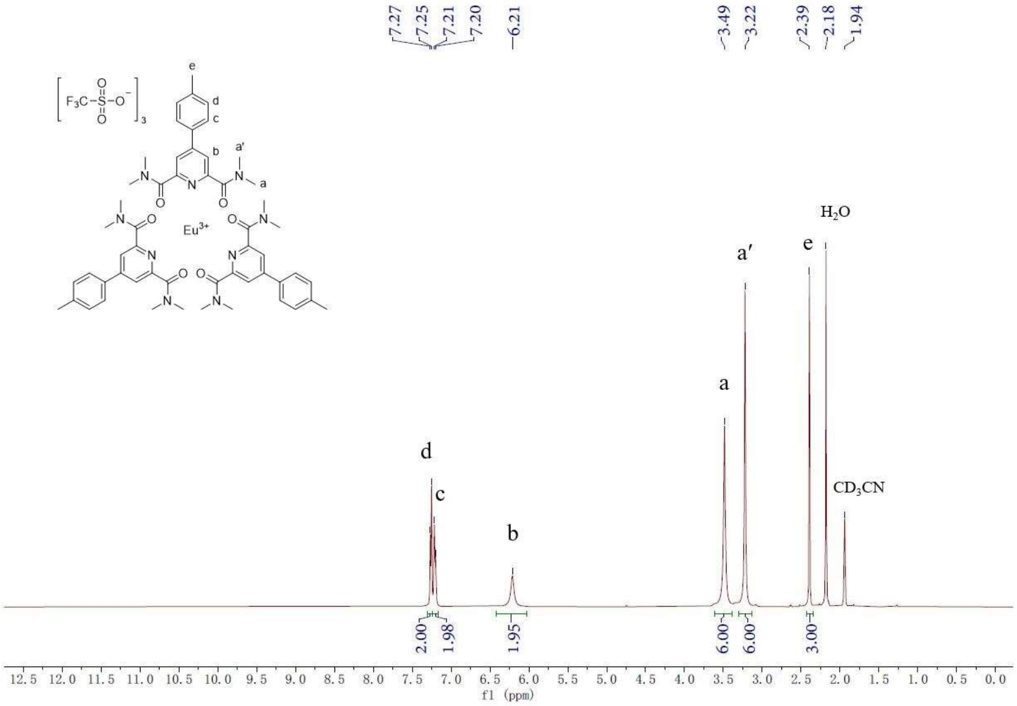

Figure S13. *^{1}H-NMR spectrum of complex **4** (500 MHz, CD_3CN, 298 K).*

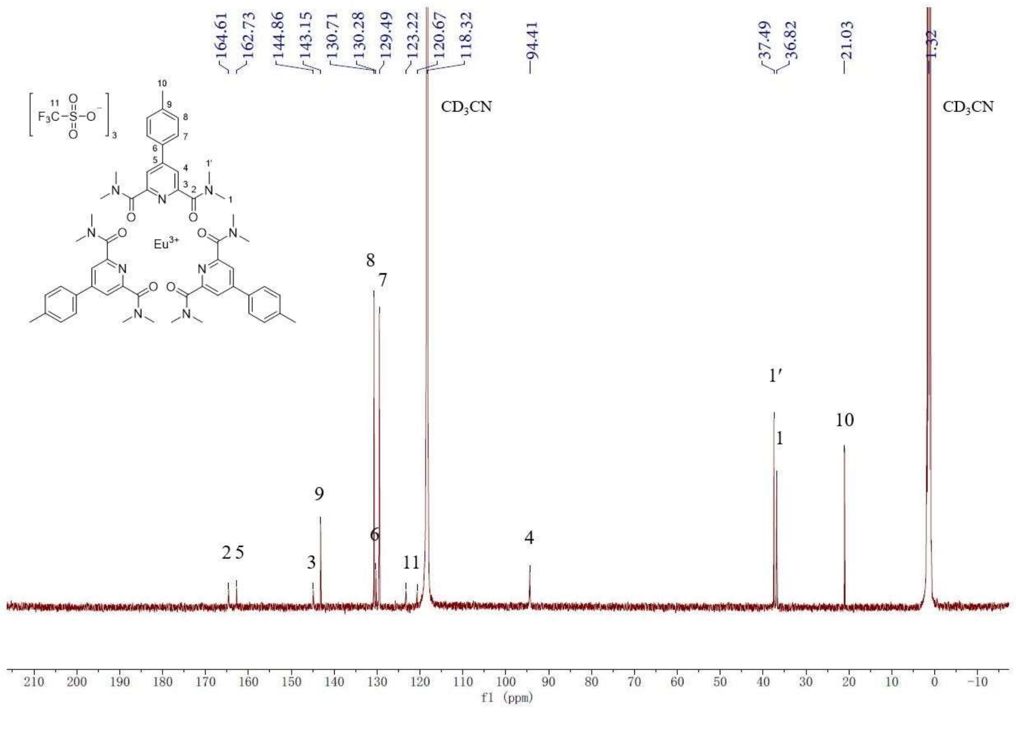

Figure S14. *^{13}C-NMR spectrum of complex **4** (126 MHz, CD_3CN, 298 K).*

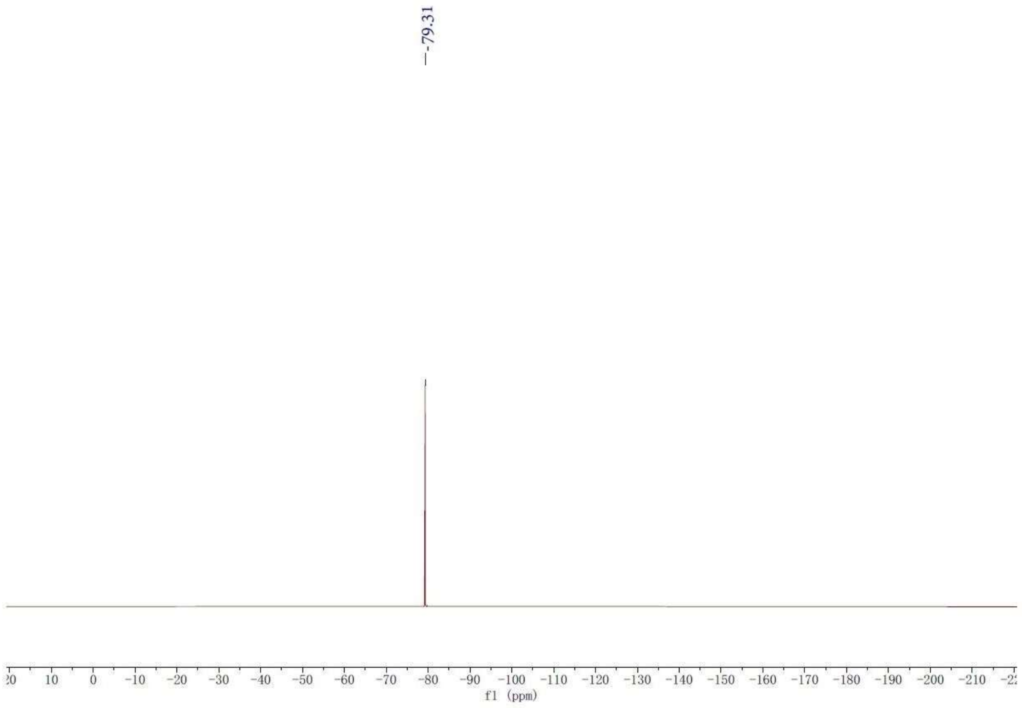

Figure S15. *^{19}F-NMR spectrum of complex* ***4*** *(471 MHz, CD_3CN, 298 K).*

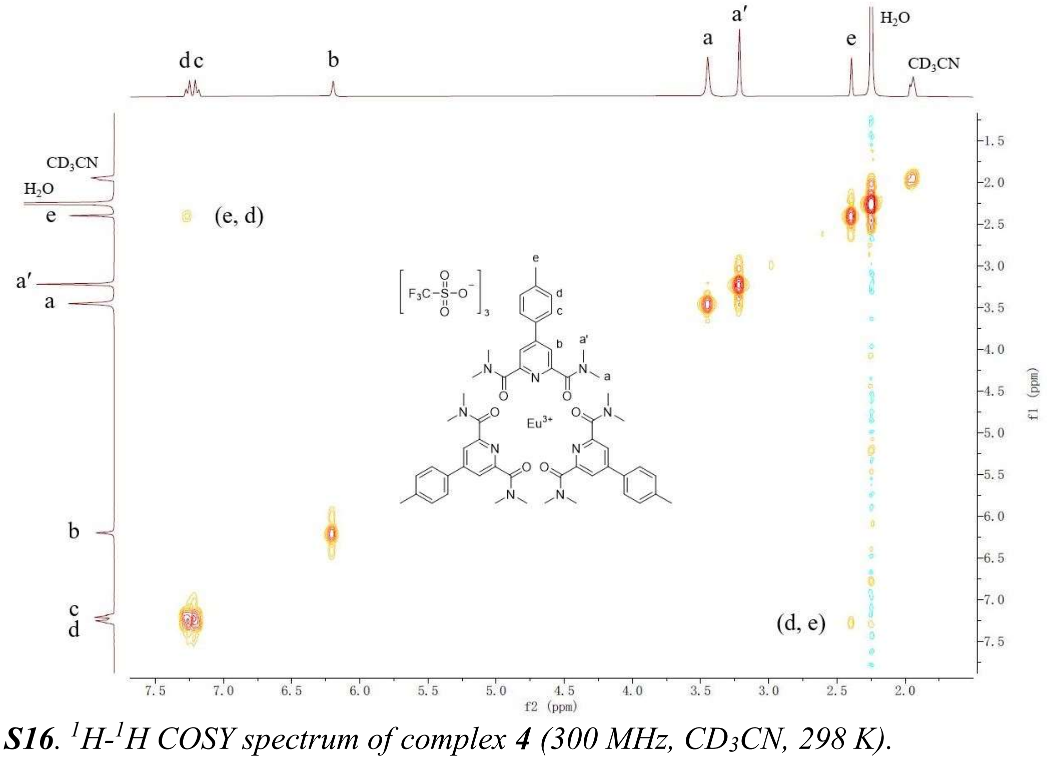

Figure S16. *^{1}H-^{1}H COSY spectrum of complex* ***4*** *(300 MHz, CD_3CN, 298 K).*

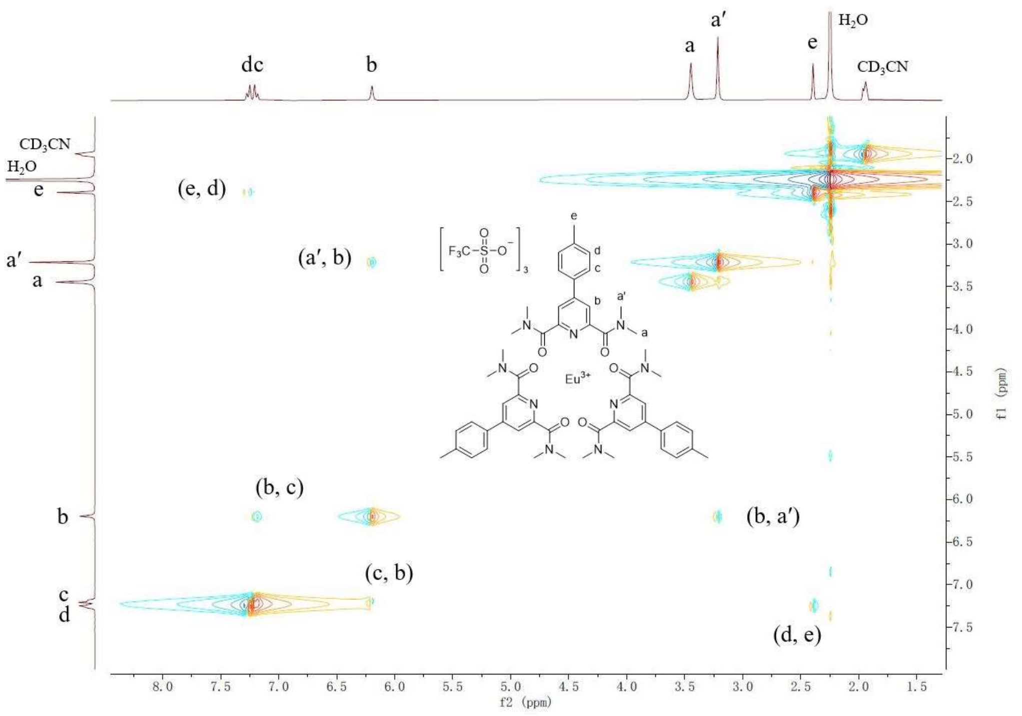

Figure S17. *^{1}H-^{1}H NOESY spectrum of complex **4** (300 MHz, CD_3CN, 298 K).*

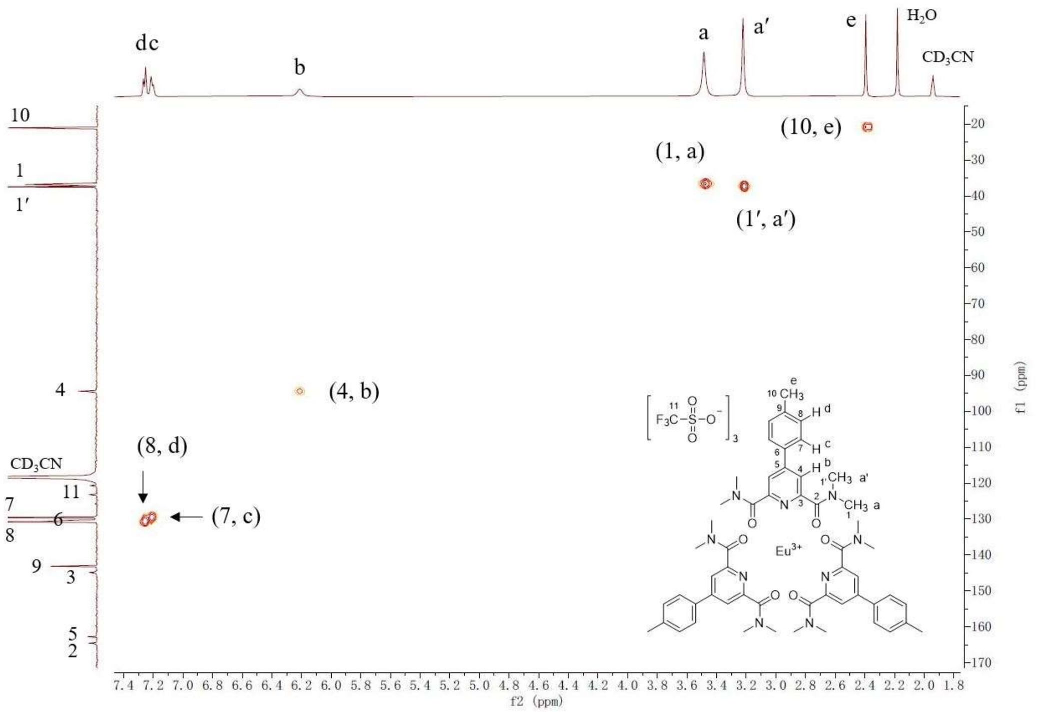

Figure S18. *^{1}H-^{13}C HSQC spectrum of complex **4** (500 MHz, CD_3CN, 298 K).*

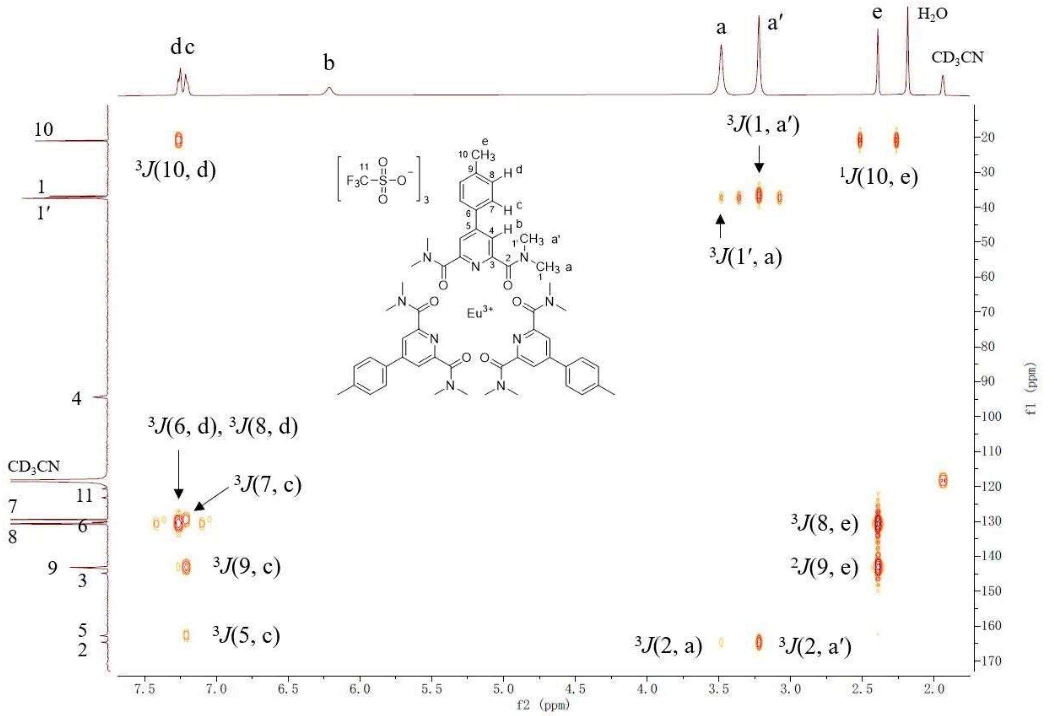

Figure S19. *^1H-^{13}C HMBC spectrum of complex **4** (500 MHz, CD_3CN, 298 K).*

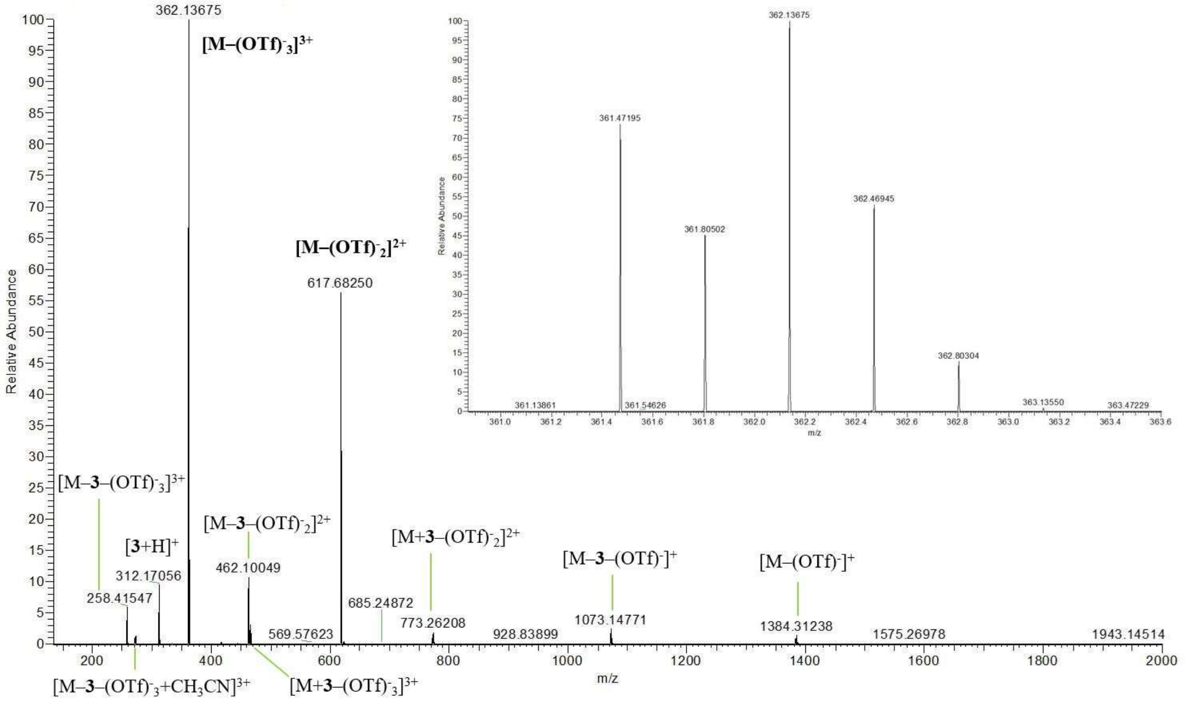

Figure S20. *HR-MS spectrum of complex **4** in CH_3CN.*

<u>Supplementary Note 1</u>. Distinguishing $[Eu(pcam)_3]^{3+}$ from $[Eu(pcam)_3X]^{2+}$

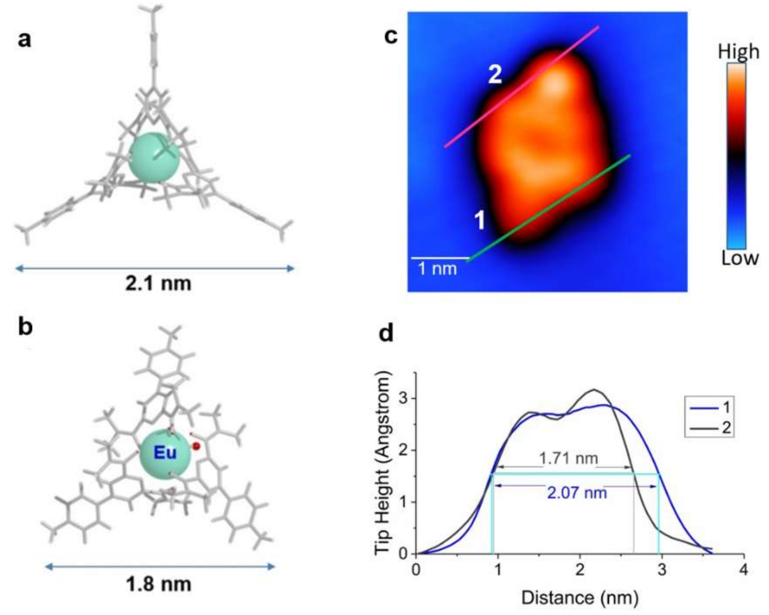

Figure S21. *Molecular Dimensions. Top views of* ***(a)*** *$[Eu(pcam)_3]^{3+}$ and* ***(b)*** *$[Eu(pcam)_3X]^{2+}$ (X = CF_3SO_3) complex.* ***(c)*** *STM image of $[Eu(pcam)_3]^{3+}$ (1) and $Eu(pcam)_3X]^{2+}$ (2).* ***(d)*** *STM line profiles along (1) and (2) shown in (c). [V_t = 0.9V, I_t = 1 x 10^{-11}A, 5K].*

$[Eu(pcam)_3]^{3+}$ has a triangular shape with a planar geometry and the average side length is 2.1 nm (Fig. S21a). In $[Eu(pcam)_3X]^{2+}$, the incorporation of a triflate ion (CF_3SO_3-) underneath the $Eu(pcam)_3$ distorts it towards a trigonal pyramid, and its side lengths are reduced (Fig. S21b). After deposition of the $Eu(pcam)_3$-$(CF_3SO_3)_3$ salt onto atomically clean Au(111) surface, isolated $[Eu(pcam)_3]^{3+}$ is not observed. However, occasionally $[Eu(pcam)_3]^{3+}$ can be found next to $[Eu(pcam)_3X]^{2+}$ complex as shown in figure S21c. Here, $[Eu(pcam)_3]^{3+}$ (1) appears as a more symmetric triangular shape while the $[Eu(pcam)_3]^{3+}$ (2) is distorted with reduced lengths as expected. The side lengths measured along the line profiles (1) and (2), 1.71 nm and 2.07 nm respectively (Fig. S21c and d), agree well with the theoretical side lengths of 1.8 nm and 2.1 nm for $[Eu(pcam)_3X]^{2+}$ and $[Eu(pcam)_3]^{3+}$, respectively (Fig. S21a and b).

A compelling evidence for the incorporation of counterion underneath the complex can be found in its electronic structure (Fig. S22). The shapes and energetic positions of the LUMOs between the $[Eu(pcam)_3]^{3+}$ and the $[Eu(pcam)_3X]^{2+}$ are drastically different. The dI/dV spectroscopy map of the LUMO of $[Eu(pcam)_3X]^{2+}$ is concentrated in one of the molecular arms only and it is located

at ~2 eV while the LUMO of $[Eu(pcam)_3]^{3+}$ is positioned on two arms and is located at ~1.6 eV (Fig. S22b, c, and d). The experimental dI/dV maps agree very well with the DFT calculated results (Fig. S22e and f). To make a clear and unequivocal distinction between the complexes with and without counterion underneath, a dI/dV spectroscopic movie is created from 8000 dI/dV maps acquired simultaneously with the STM image shown in Fig. S22a (see Supplementary Movie 1). Here, dI/dV spectroscopic maps are acquired at every 1 mV interval between +2000 mV and -2000 mV, and then repeated back to +2000 mV to ensure reproducibility. This movie clearly reveals the differences in orbital shapes between 2000 mV and 1200 mV where the LUMOs of the two complexes (Fig. S22b and c) are located.

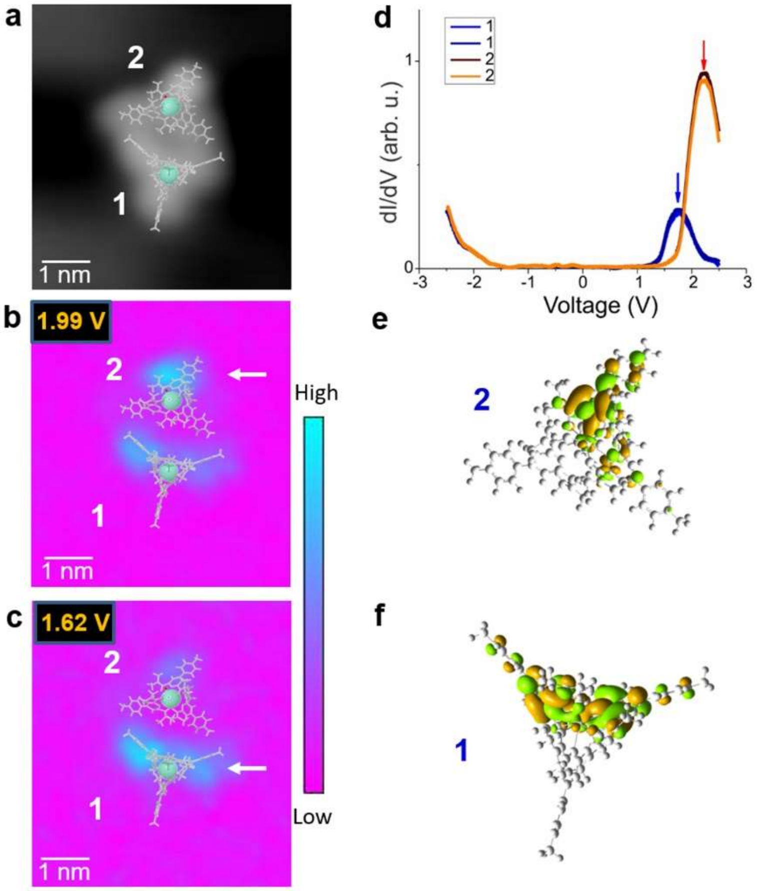

Figure S22. *Comparison between the LUMOs of $[Eu(pcam)_3]^{3+}$, and $[Eu(pcam)_3X]^{2+}$ complexes.* ***(a)*** *STM image of $[Eu(pcam)_3]^{3+}$ (1), and $[Eu(pcam)_3X]^{2+}$ (2) [$V_t = 0.9V$, $I_t = 1 \times 10^{-11}A$, 5K].* ***(b), (c)*** *Corresponding dI/dV maps at 1.99 V and 1.62 V showing the LUMO of $[Eu(pcam)_3X]^{2+}$ and $[Eu(pcam)_3]^{3+}$, respectively. The models of complexes are overlaid on the STM image (a) and spectroscopy maps (b, c).* ***(d)*** *Point dI/dV spectra acquired over the centre of the $[Eu(pcam)_3]^{3+}$ (blue) and $[Eu(pcam)_3X]^{2+}$ (brown/orange), respectively. The corresponding LUMOs are indicated with arrows.* ***(e)*** *Calculated LUMO orbital of $[Eu(pcam)_3X]^{2+}$, and* ***(f)*** *$[Eu(pcam)_3]^{3+}$.*

Supplementary Note 2. Calculated HOMO and LUMO Orbitals of the Complexes

The highest occupied molecular orbitals (HOMO) and lowest unoccupied molecular orbital (LUMO) were calculated by using single point DFT method in gas phase. Single point DFT calculations were performed by using Gaussian 16 software package[1] on the geometries optimized by VASP software[2] to visualize molecular orbitals of $[Eu(pcam)_3]^{3+}$, $[Eu(pcam)_3X]^{2+}$, and $[Eu(pcam)_3X_2]^{+}$ complexes. PBE1PBE[3,4] density functional was used in these calculations. All the atoms including Eu center were described with def2-TZVP basis set[5,6]. The HOMO and LUMO orbitals were visualized using Gaussview 5.0[7].

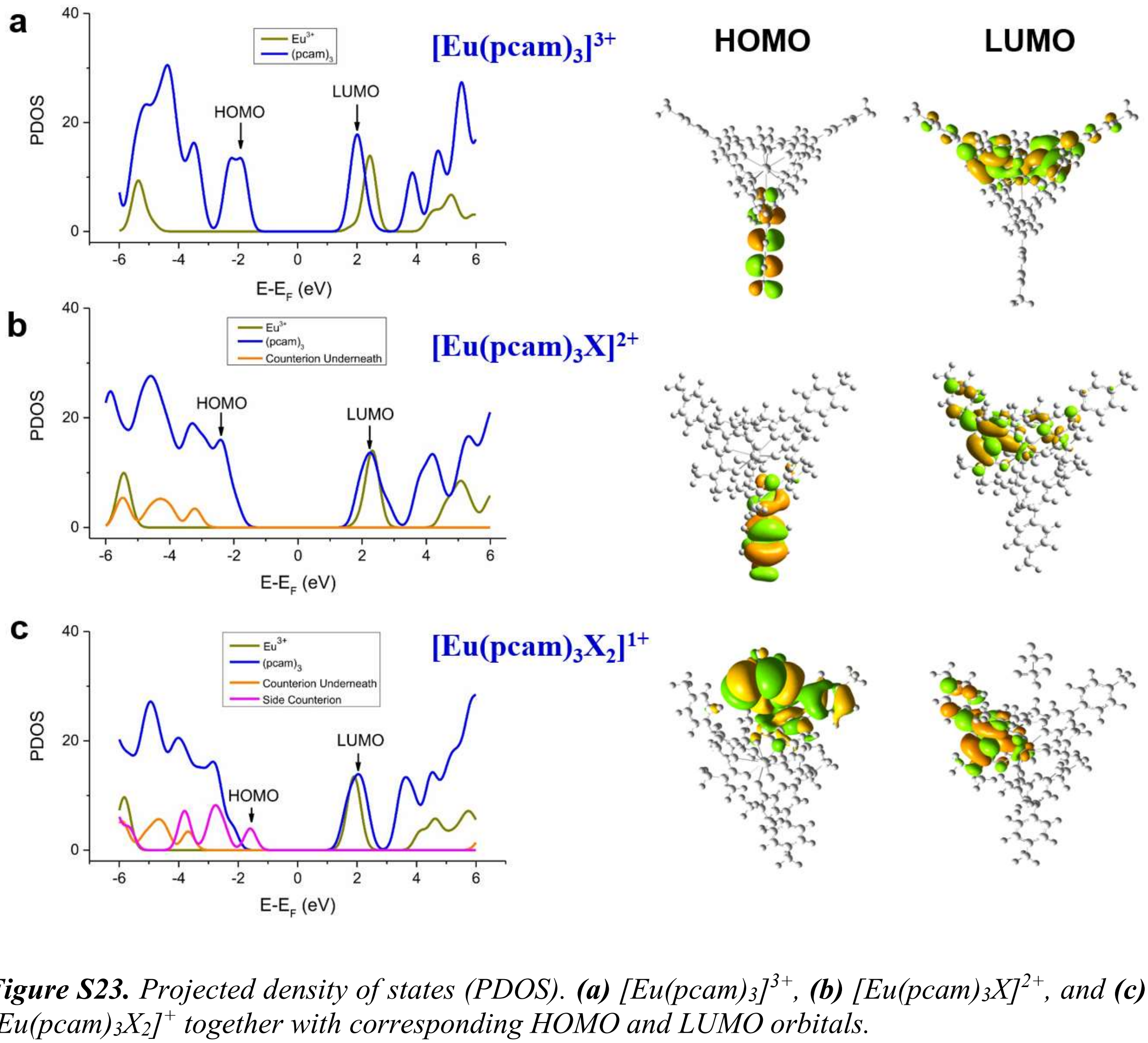

Figure S23. *Projected density of states (PDOS).* ***(a)*** *$[Eu(pcam)_3]^{3+}$,* ***(b)*** *$[Eu(pcam)_3X]^{2+}$, and* ***(c)*** *$[Eu(pcam)_3X_2]^{+}$ together with corresponding HOMO and LUMO orbitals.*

The calculated orbital shapes can be directly compared with the STM dI/dV spectroscopic maps (see Supplementary Note 1, and 4). The energetic positions of the HOMO and LUMO are also in qualitative agreement with the experiment, indicating that their electronic structures do not alter significantly after adsorption on Au(111) surface (see Supplementary Table S1). Geometrically relaxed calculations reveal that the electronic structures of $[Eu(pcam)_3]^{3+}$, $[Eu(pcam)_3X]^{2+}$ and $[Eu(pcam)_3X_2]^{+}$ complexes are very different.

Supplementary Table S1. *HOMO and LUMO of the complexes.*

	HOMO (eV)	LUMO (eV)	Gap (Ev)
$[Eu(pcam)_3]^{3+}$	-1.93	1.99	3.92
$[Eu(pcam)_3X]^{2+}$	-2.41	2.25	4.66
$[Eu(pcam)_3X_2]^{+}$	-1.6	1.89	3.49

<u>Supplementary Note 3</u>. Calculated Density of States

The electronic structures of the complexes are also calculated by DFT method using VASP code (see methods section in the main text) for gas phase as well as the ones adsorb on Au(111). The density of states (DOS) of the Eu(pcam)$_3$ and the counterion underneath from the complex [Eu(pcam)$_3$X$_2$]$^+$ adsorbed on Au(111) surface are provided in Fig. 3d, and 3e in the main text. The DOS for the side counterion from the complex [Eu(pcam)$_3$X$_2$]$^+$ adsorbed on Au(111) is shown below (Fig. S24a). The gas phase DOS for Eu(pcam)$_3$, counterion underneath, and the side counterion of the complex from the complex [Eu(pcam)$_3$X$_2$]$^+$ are shown in Fig. S24c, d, and e, respectively.

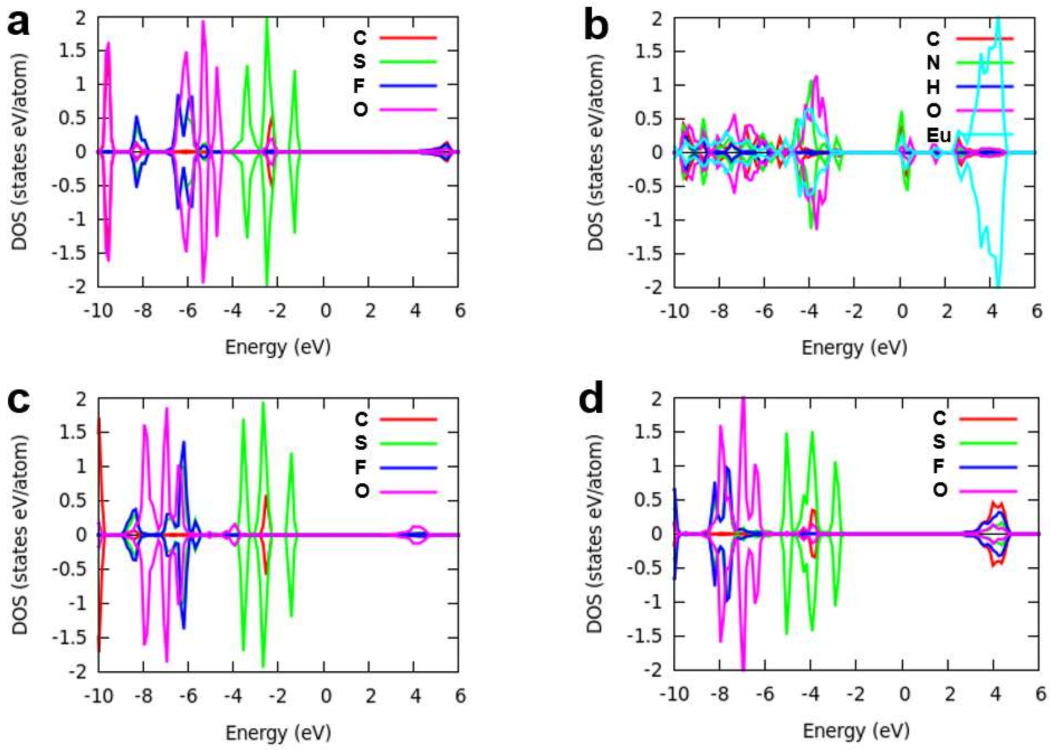

Figure S24. *Density of states.* ***(a)*** *Calculated DOS of side counterion from a complex [Eu(pcam)$_3$X$_2$]$^+$ adsorbs on Au(111).* ***(b)*** *DOS of [Eu(pcam)$_3$]$^{3+}$ in gas phase.* ***(c)*** *DOS of side counterion from a complex [Eu(pcam)$_3$X$_2$]$^+$ in gas phase.* ***(d)*** *DOS of counterion underneath [Eu(pcam)$_3$X$_2$]$^+$ in gas phase.*

Supplementary Note 4. dI/dV Spectroscopy Maps of [Eu(pcam)$_3$X$_2$]$^+$ Complex

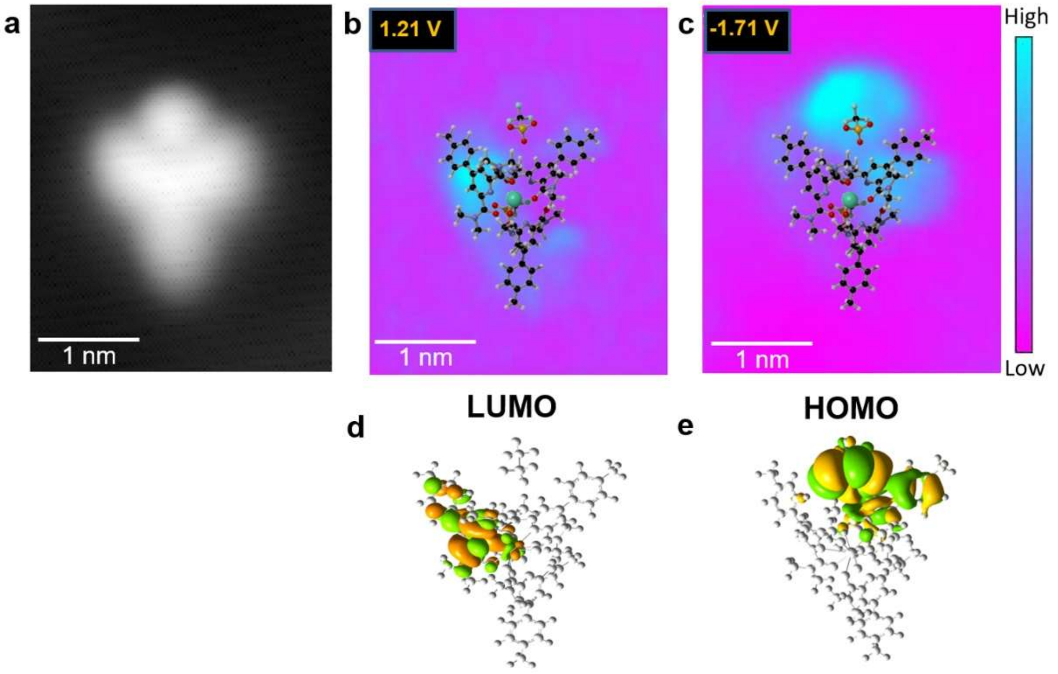

Figure S25. *Molecular orbitals of [Eu(pcam)$_3$X$_2$]$^+$ complex.* ***(a)*** *STM image of [Eu(pcam)$_3$X$_2$]$^+$. [V_t = 0.5V, I_t = 1 x 10^{-11}A, 5K]. Corresponding dI/dV maps at 1.21 V* ***(b)*** *and at -1.71 V* ***(c)*** *belong to LUMO and HOMO of the complex, respectively. A complex model is overlaid to guide the spatial locations of the orbitals in (b) and (c).* ***(d)*** *and* ***(e)*** *are calculated LUMO and HOMO of the complex, respectively.*

The measured dI/dV tunneling spectroscopic maps of the [Eu(pcam)$_3$X$_2$]$^+$ complex and corresponding calculated orbitals are presented in figure S25. The LUMO is captured at +1.21 V, which shows that the orbital is centered on the left arm of the complex (Fig. S25b) in agreement with the calculated LUMO (Fig. S25d). The HOMO is captured at ~ -1.71 V, which shows the orbital location as the side counterion while a reduced charge contribution can also be found in the right arm and periphery of the Eu ion (Fig. S25c). This is also in agreement with the calculated HOMO of the complex (Fig. S25e). The energetic positions of the HOMO and LUMO orbitals are shown in Fig. 3b and 3c in the main text.

Supplementary Note 5. Calculations of Valence Charges in the Complexes

In the gas phase, the Eu ion in $[Eu(pcam)_3]^{3+}$ has a +3e electric charge while each triflate counterion has a -1e charge. Therefore, $[Eu(pcam)_3X]^{2+}$ complex should have a net charge of +2e while the complex with an additional side counterion, $[Eu(pcam)_3X_2]^{+}$ should have a net charge of +1e.

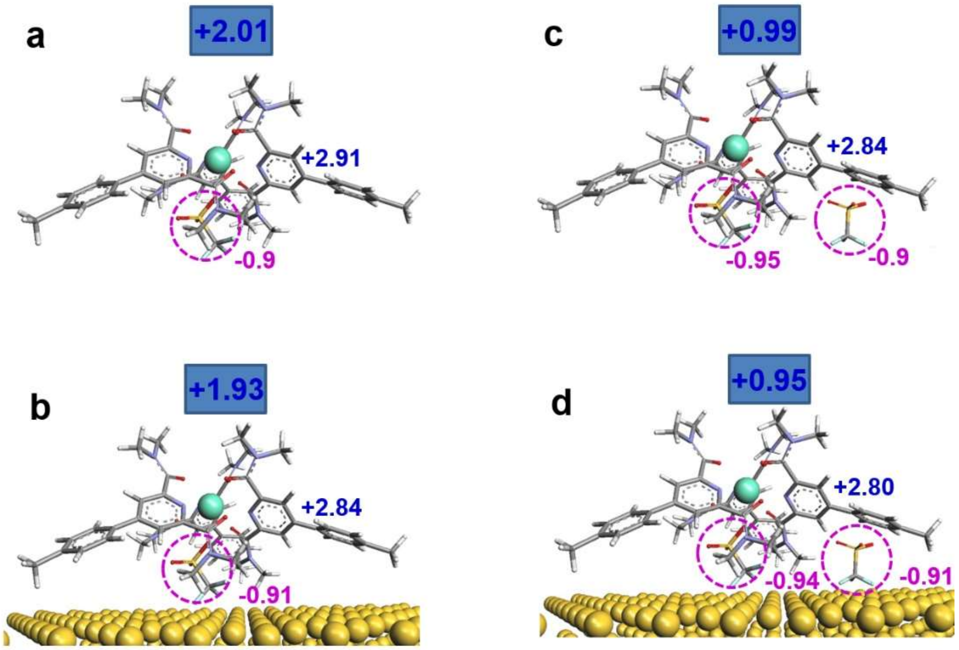

Figure S26. *Calculated charges for $[Eu(pcam)_3X]^{2+}$ complex in the gas phase* ***(a)*** *and on Au(111)* ***(b)****, and for the $[Eu(pcam)_3X_2]^{+}$ complex in the gas phase* ***(c)*** *and on Au(111)* ***(d)****. Positive charges are for the $Eu(pcam)_3$ and the negative charges are in the counterions. The net charges of the complexes are shown in the box above the complex structures. The unit of charges are in 'e'.*

In order to analyze the charges of the complexes on Au(111) surface, we have performed calculations where the valence charges were obtained using a computation method developed by the Henkelman group[8,9,10], by partitioning a charge density grid into Bader volumes. Charges enclosed within the Bader volume are a good approximation of the electronic charges of an atom[4-6,11]. The calculations were performed on both types of complexes in the gas phase and on the Au(111) surface (Fig. S26). The results are summarized in Supplementary Table S2.

The calculated results for both complexes in the gas phase agree very well with the expected values. For the $[Eu(pcam)_3X]^{2+}$ (type 'A' complex), the $Eu(pcam)_3$ unit has a charge of +2.91e while the counterion underneath has a charge of -0.9e. Therefore, the total charges of $[Eu(pcam)_3X]^{2+}$ complex is +2.01e, which agrees with the expected value of +2e charge for this complex (Fig. S26a, and Supplementary Table S2). For $[Eu(pcam)_3X_2]^{+}$ (type 'B' complex), the

$Eu(pcam)_3$ unit has +2.84e charge while the charges for the two counterions are -0.95e for the one underneath the molecule, and -0.9e for the side counterion, respectively. Thus, the total charge for the $[Eu(pcam)_3X_2]^+$ complex is +0.99e, which again agrees with the expected value of +1e (Fig. S26c, and Supplementary Table S2).

Supplementary Table S2. *The valence charges of Eu(pacam)₃ and triflate counterions*

Complex	Total valence charge of complexes		Decomposition of valence charges			
			$Eu(pcam)_3$		$CF_3SO_3^-$ counterions	
	Gas phase	On Au(111)	Gas phase	On Au(111)	Gas phase	On Au(111)
$[Eu(pcam)_3X]^{2+}$	+2.01e	+1.93e	+2.91e	+2.84e	-0.90e	-0.91e
$[Eu(pcam)_3X_2]^+$	+0.99e	+0.95e	2.84e	+2.80e	-0.95e<br>-0.90e	-0.94e<br>-0.91e

Next, the calculations were performed for the complexes adsorbed on a three layer Au(111) slab. For complex $[Eu(pcam)_3X]^{2+}$, the charge of $Eu(pcam)_3$ is slightly reduced to +2.84e while that of the counterion is slightly increased to 0.91e (Fig. S26b, and Supplementary Table S2). The net charge of the complex on Au(111) is +1.93e. Similarly, for complex $[Eu(pcam)_3X_2]^+$, $Eu(pcam)_3$ has a slightly reduced charge of +2.80e while the two counterions have charges of -0.94e and -0.91e, respectively. Here again the total charge of the complex on Au(111) is slightly reduced to +0.95e (Fig. S26d, and Supplementary Table S2).

The slight reduction of total charge from +2.01e to +1.93e in complex $[Eu(pcam)_3X]^{2+}$ after adsorption on Au(111) surface indicates that there is only a negligible amount of charge transfer from the substrate. The complex $[Eu(pcam)_3X_2]^+$ exhibits a similar trend as only a small change in the total valence charge occurs, from +0.99e in the gas phase to +0.95e on Au(111). These results clearly suggest no significant charge transfer from the Au(111) substrate to both $Eu(pcam)_3$ unit and counterions of the complexes. Thus, the net charge of the complex is intact on the Au(111) substrate. This result agrees with the NEXAFS measurement using synchrotron X-rays scanning tunnelling microscopy (Fig. 3f) and controlled directional rotation of the $Eu(pcam)_3X_2]^+$ (Fig. 5a, 5b, and 5c) in the main text.

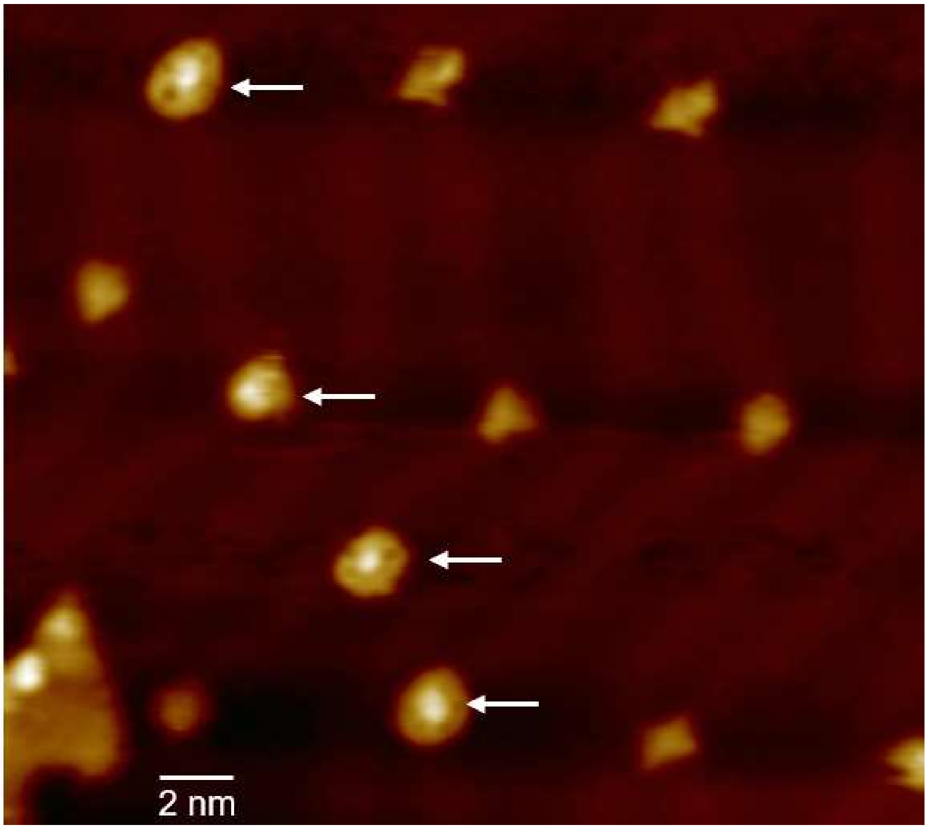

Figure S27. *A large area STM image showing thermally excited rotation of complexes (indicated with arrows). [V_t = 1.0V, I_t = 3 x 10^{-11}A, 100K].*

<u>Supplementary Note 6</u>. Controlled Rotation of Charged Rare-Earth Complexes

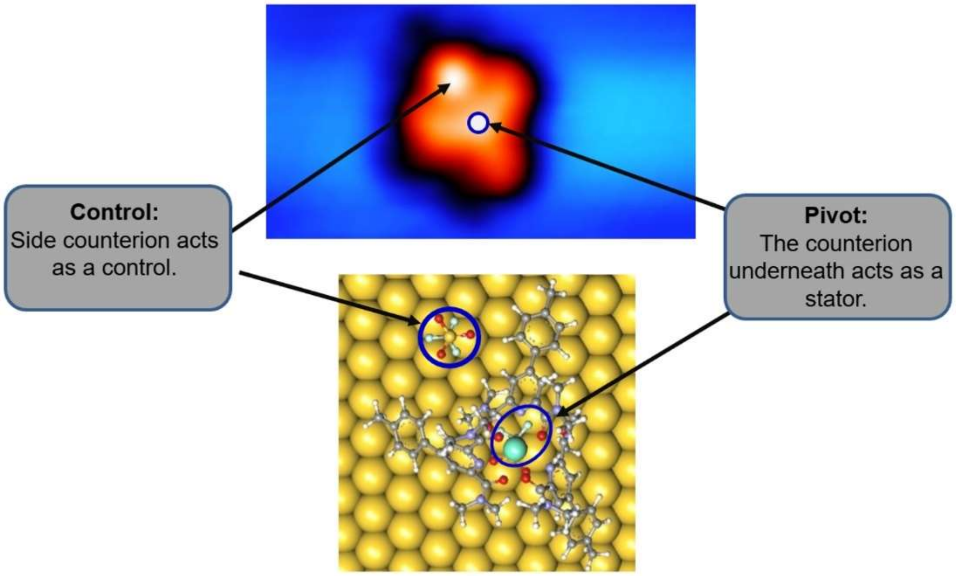

Figure S28. *Key components of the complex for controlled rotations.*

The Eu(pcam)$_3$ unit of the [Eu(pcam)$_3$X$_2$]$^+$ rotates using a triflate counterion (CF_3SO_3-) located underneath as the pivot. The negatively charged counterion at the side acts as a control unit for directional rotation and angle selection. All the components, the pivot (CF_3SO_3-), rotator (Eu(pcam)$_3$) and the control counterion (CF_3SO_3-) are held together by electrostatic interactions without chemical bonding between them.

For a controlled rotation, the STM tip is positioned next to the counterion, and a negative electric field is applied by ramping the bias (positive sample bias). When a negative electric field is applied by the tip, then the negatively charged counterion is pushed away in the opposite direction of the tip when the threshold electric field exceeds the barrier. This trigger rotation suing the triflate counterion underneath the complex as a pivot. By selecting the tip position opposite to the rotation (Fig. S29), both clockwise and anticlockwise rotations can be induced with 100% precision. When the tip applies a positive electric field, the attraction between the negatively charged side counterion and the tip results in an attachment of the complex to the tip, and the STM imaging condition is destroyed. Thus, only negative electric field of the tip can be used to control the rotation of the complexes.

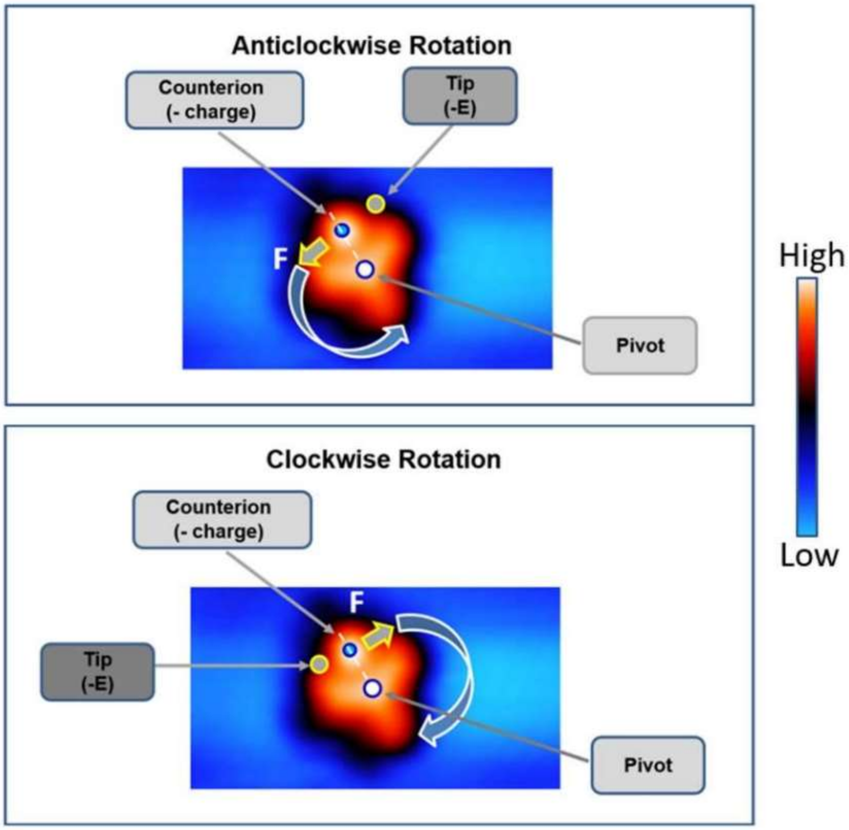

Figure S29. *Controlled directional rotation scheme.*

To establish a deterministic control over the rotation direction, we have performed 709 controlled directional rotations, and 100% control of the rotation direction is achieved. Unlike the rotation of the complex by positioning the STM tip above the center (Fig. 4 in the main text), this controlled rotation procedure can occasionally cause a slight lateral displacement since it pushes from the side of the complex via Coulomb repulsion. When the complex laterally displaces between fcc and hcp sites of Au(111) during the process, other rotation angles such as 60° or 180° can also be induced although majority of the rotations are in 120° angle. STM image frames corresponding to 523 rotation events in eight consecutive rotation series are provided in Fig. S31 to S54 where 328 rotations are in 120° (~64 %) while 129 rotations are in 60° (~25 %). 100% of the rotations follow the intended rotational directions, i.e. clockwise or anticlockwise directions.

Figure S30 provides the explanations of the markings on STM images in rotation sequences. Each rectangular frame in the rotation sequences (Fig. S31-S54) includes two STM images, one for the

initial stage (left image) and the other for the final stage after rotation (right image). The final image in the preceding frame is used as an initial image in the consecutive frame (Fig.S30). The tip positions are automatically recorded by the STM program, and additional markings, a brown oval for clockwise and a yellow oval for anticlockwise rotations at the initial images, are added for eye guidance. At the final STM image (the right image in the frame), the rotation direction is indicated with curved arrows, which trace the rotation of the complex head, where the brown curved arrows mark clockwise rotation and the yellow curved arrows indicate the anticlockwise rotation, respectively.

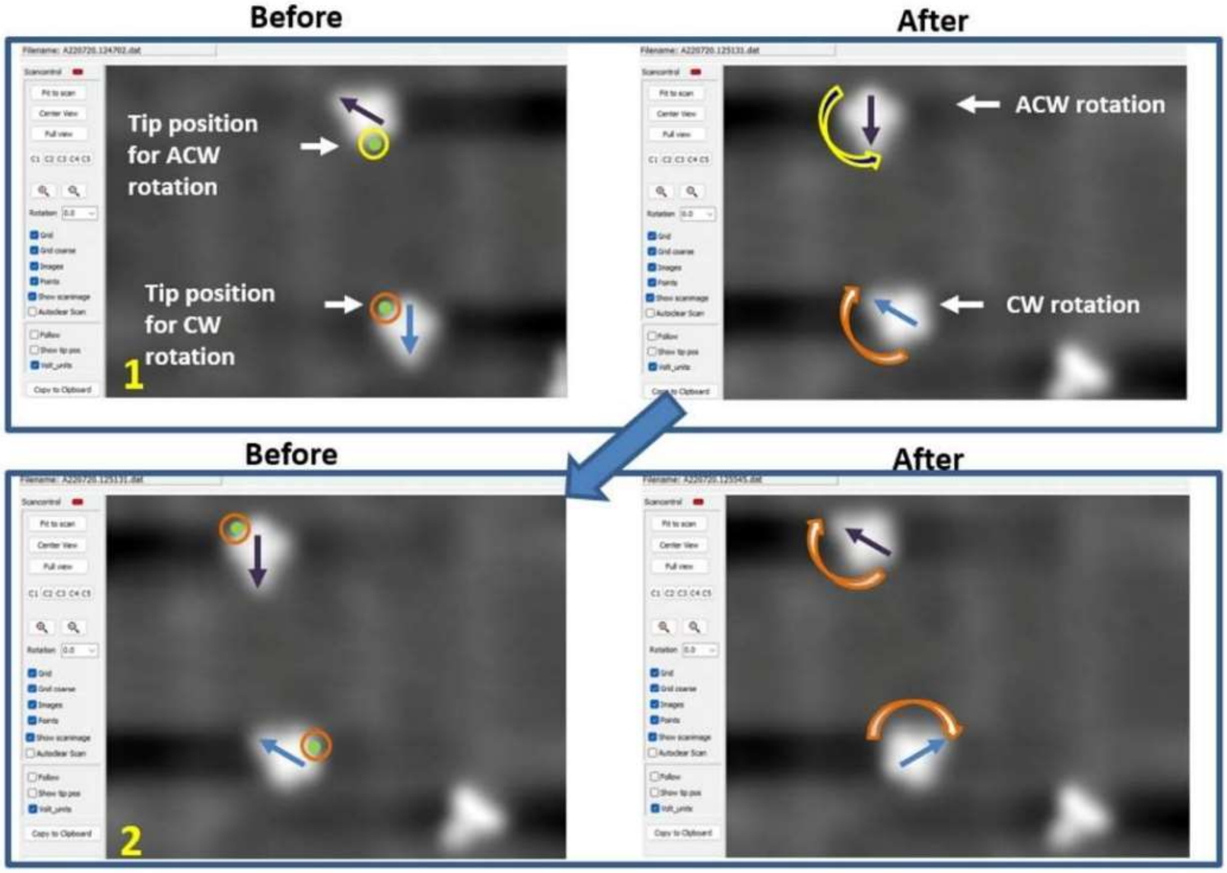

Figure S30. *Explanations of markings in the rotation sequence. Two motor complexes are shown with brown and light blue arrows, which also indicate the directions of the complexes. STM tip positions are shown with brown (clockwise) and yellow (anticlockwise) ovals in initial images. Rotation directions are shown in the final images with curved arrows; blue for clockwise (CW) and yellow for anticlockwise (ACW), respectively. The curved arrows give eye guidance for the rotation direction of the arrowheads of the complexes. Final STM image from the previous frame is used as the initial frame for the next rotation and the frames are numbered sequentially.* `

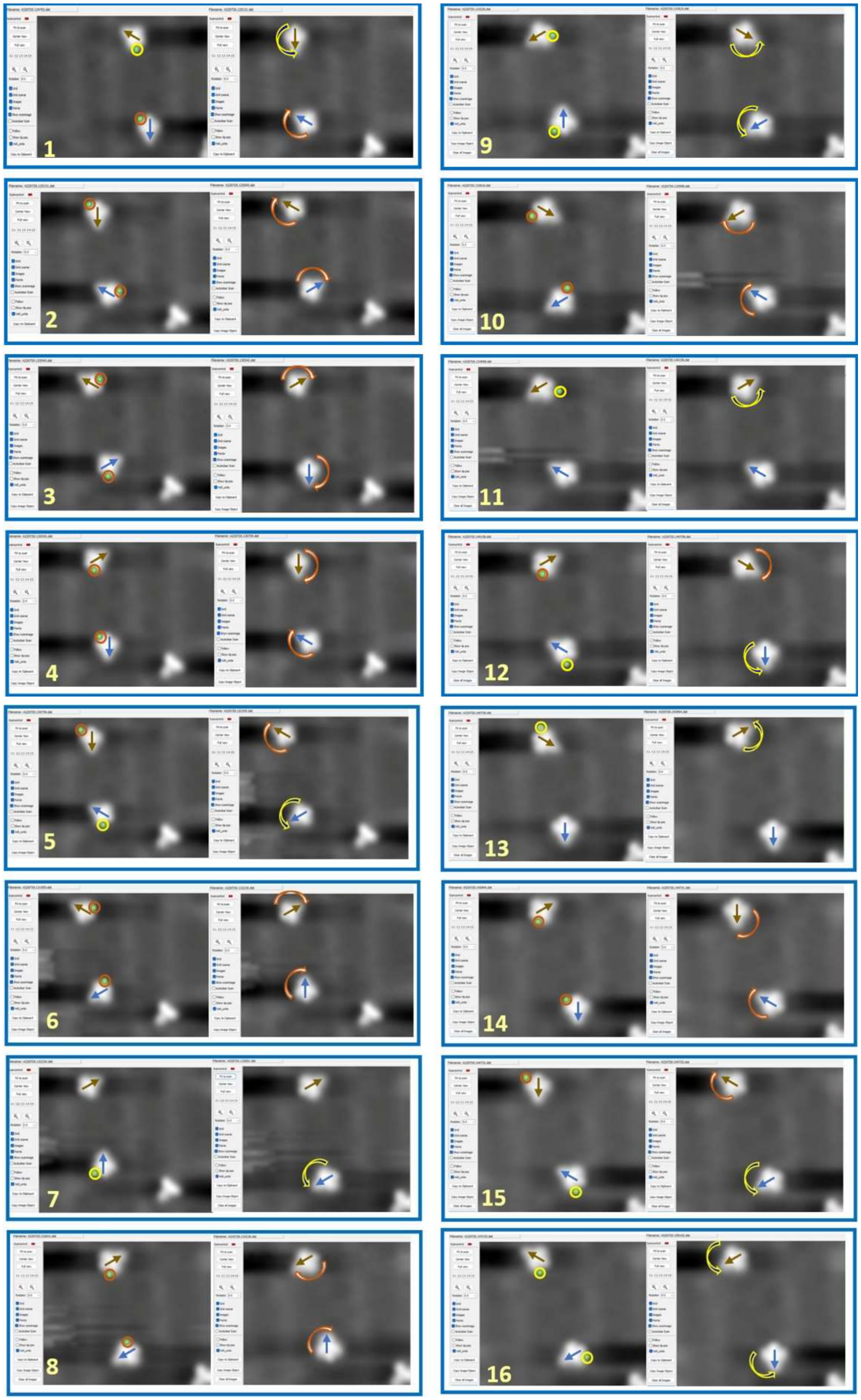

Figure S31. *Rotation sequence 1*

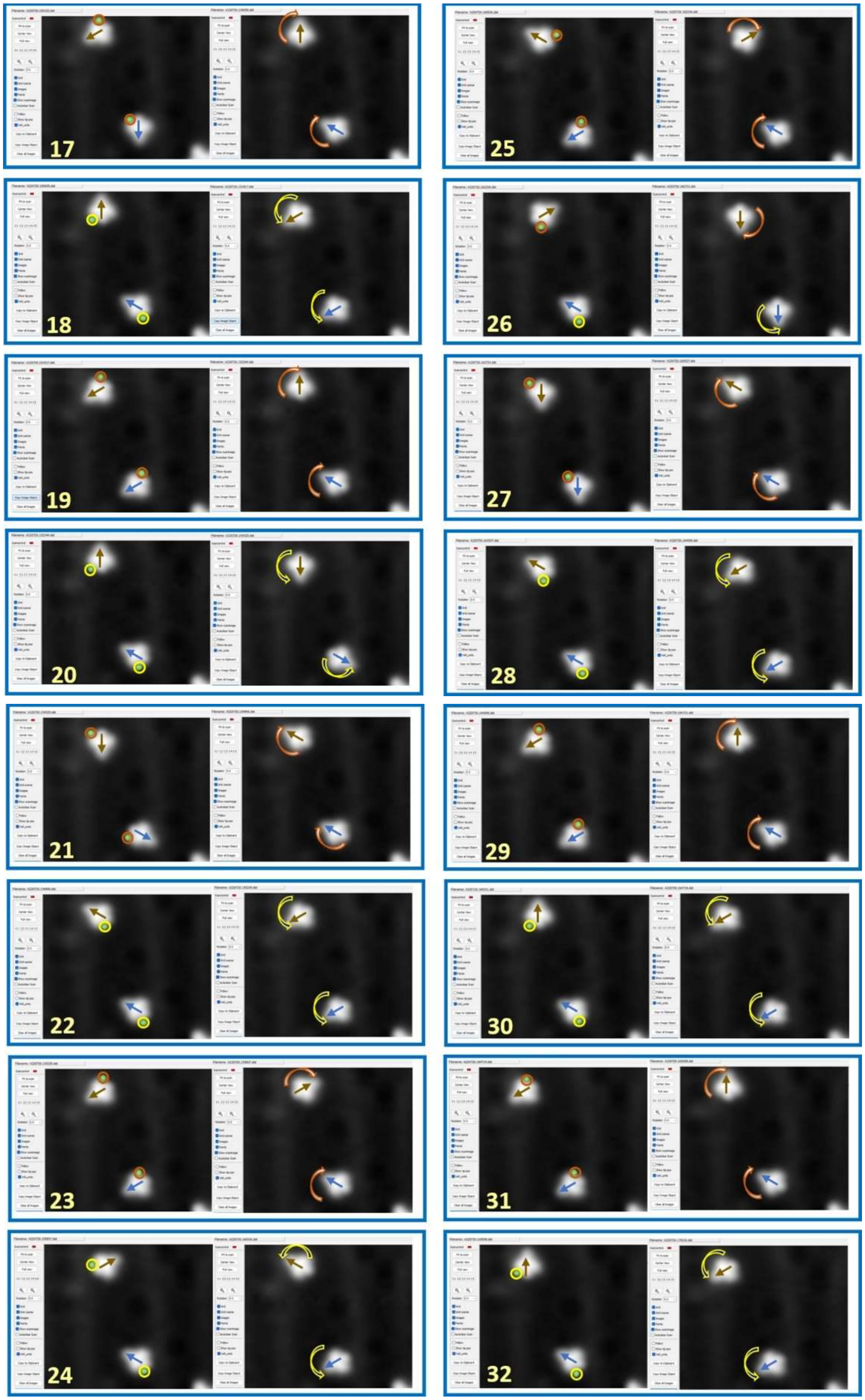

Figure S32. *Rotation sequence 1 continued.*

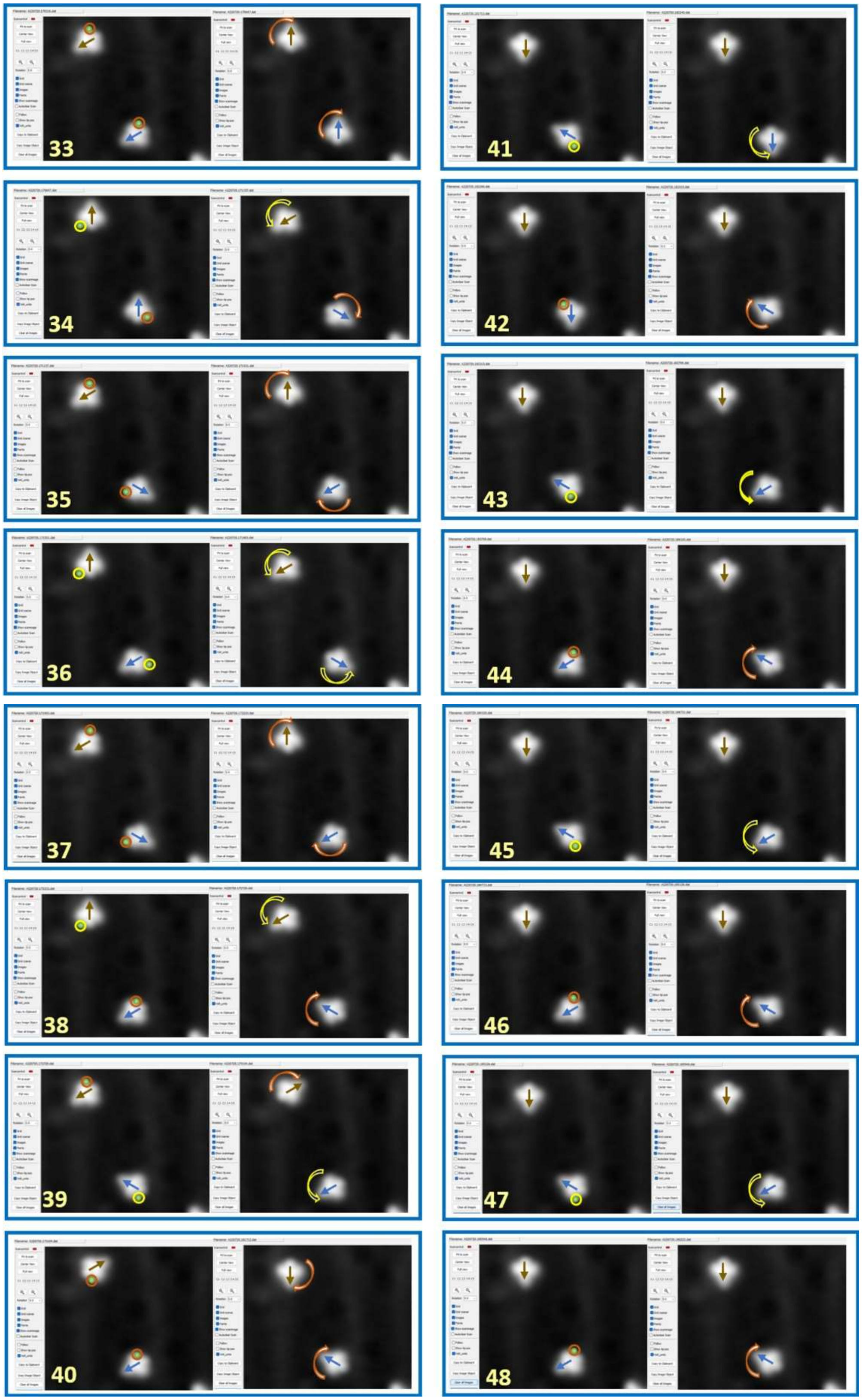

Figure S33. *Rotation sequence 1 continued.*

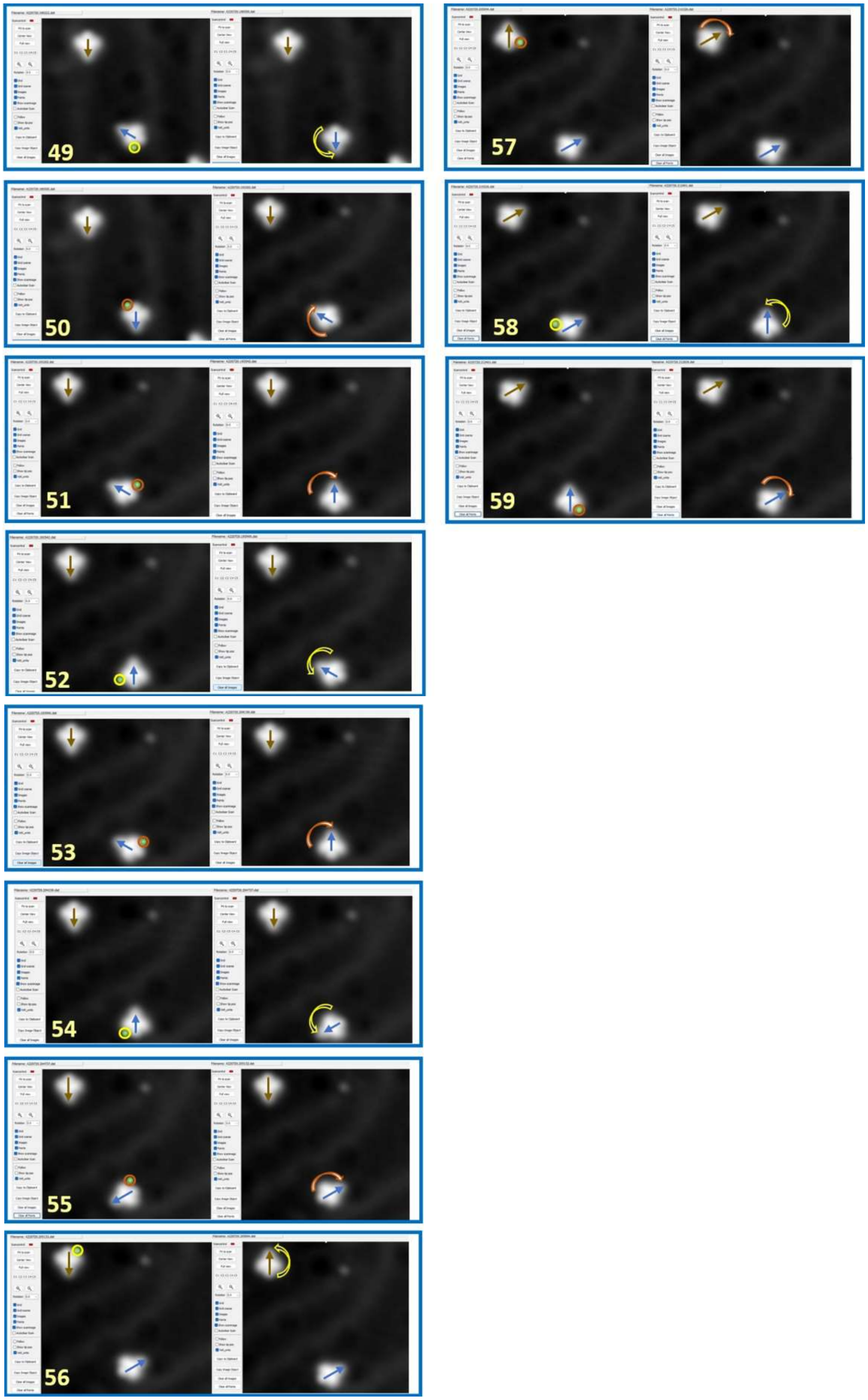

Figure S34. *Rotation sequence 1 continued.*

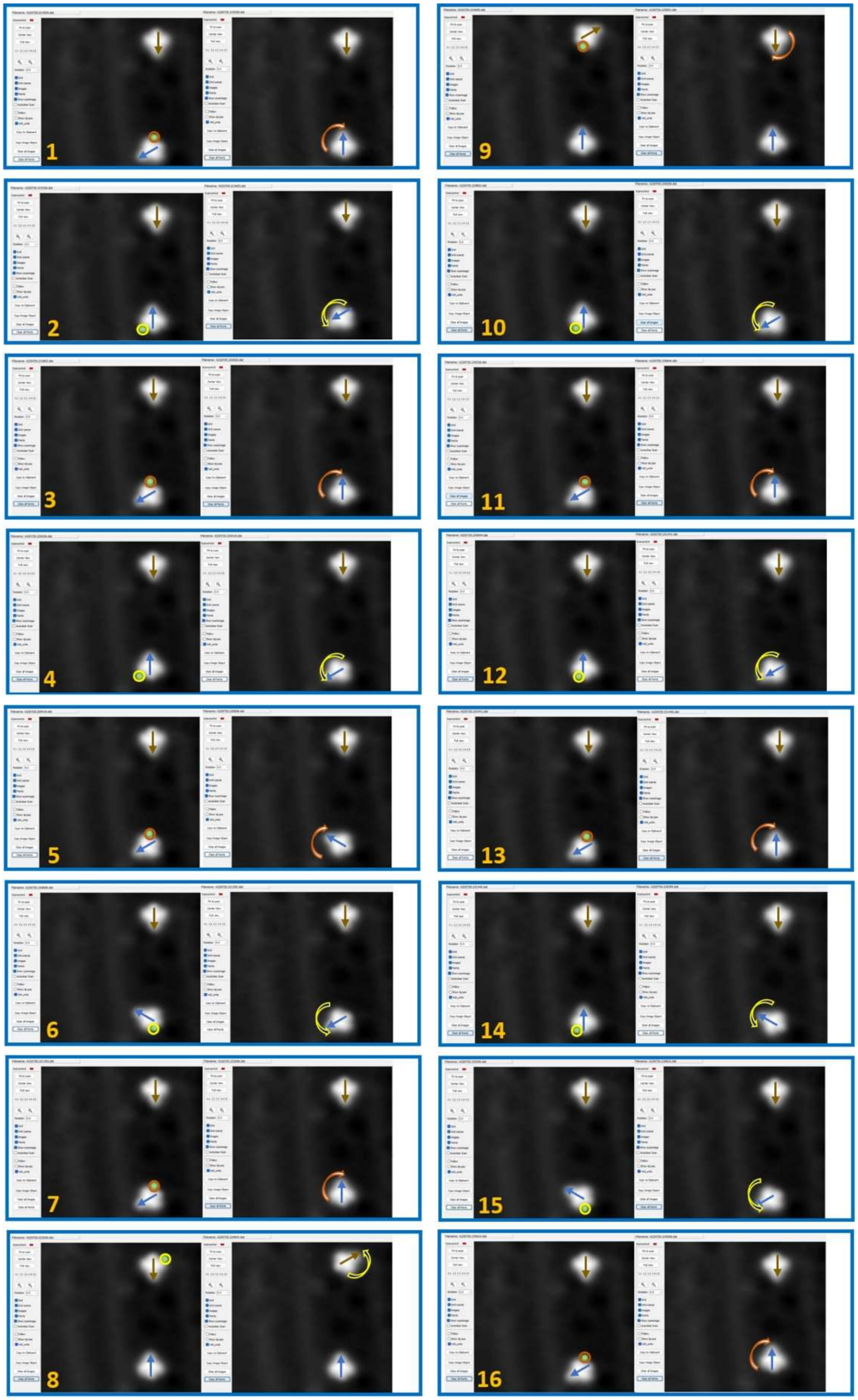

Figure S35. *Rotation sequence 2.*

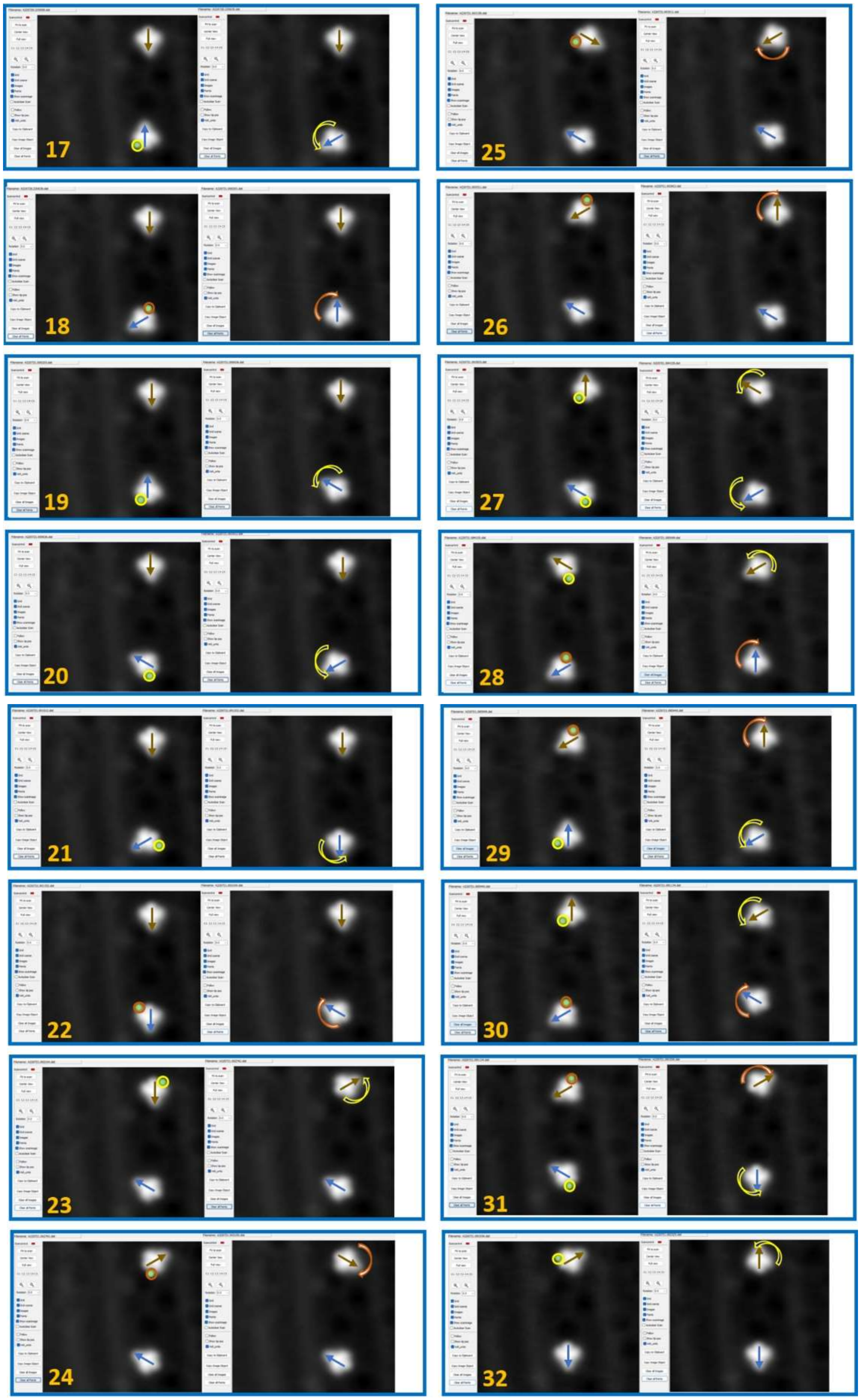

Figure S36. *Rotation sequence 2 continued.*

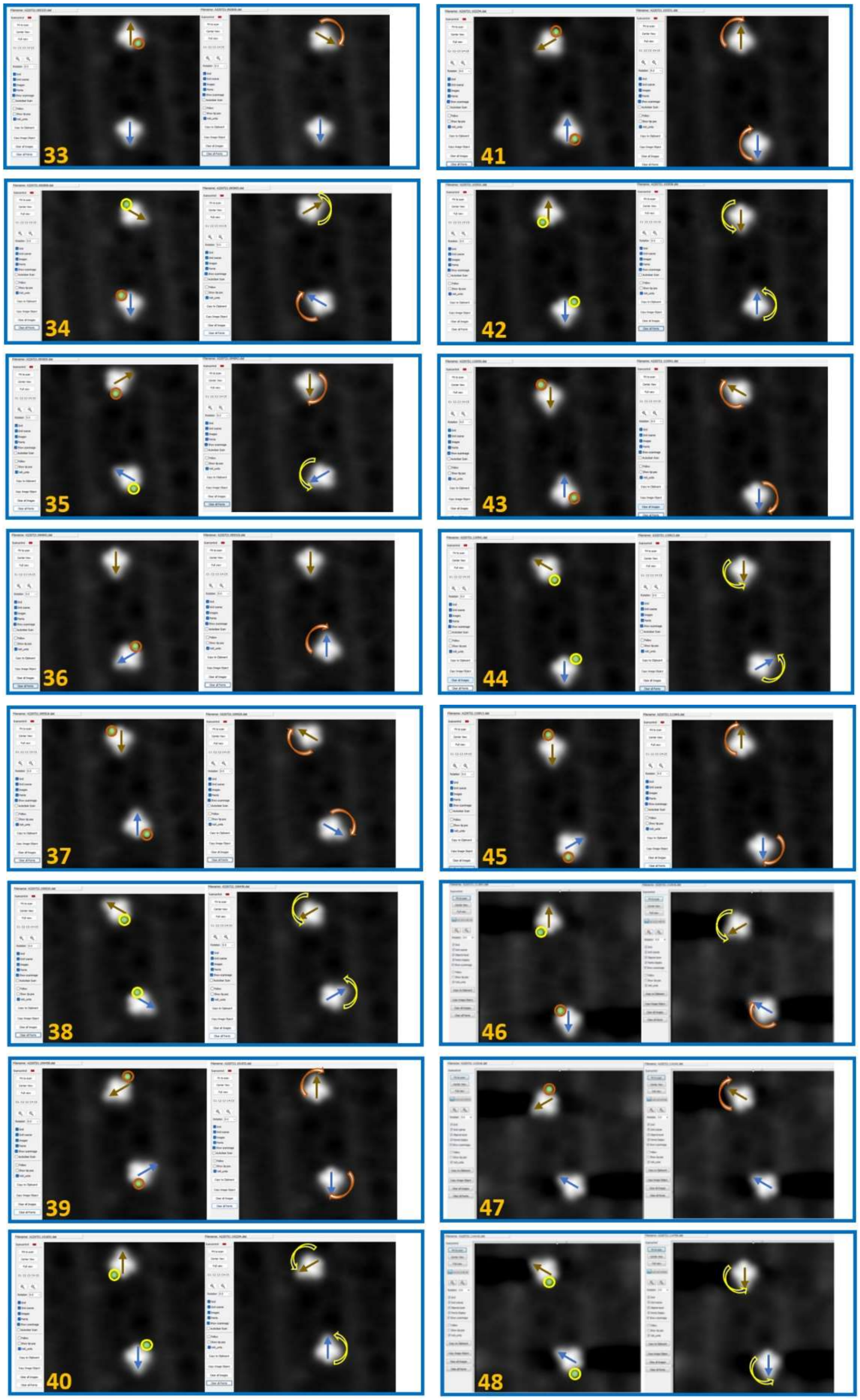

Figure S37. *Rotation sequence 2 continued.*

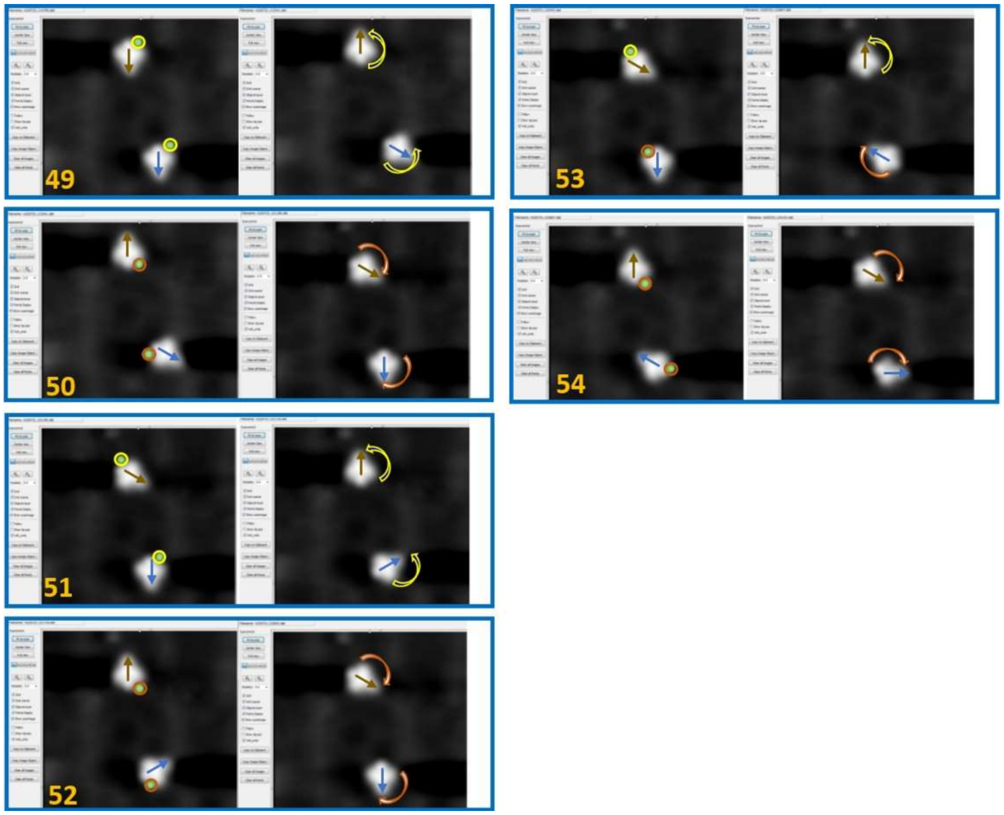

Figure S38. *Rotation sequence 2 continued.*

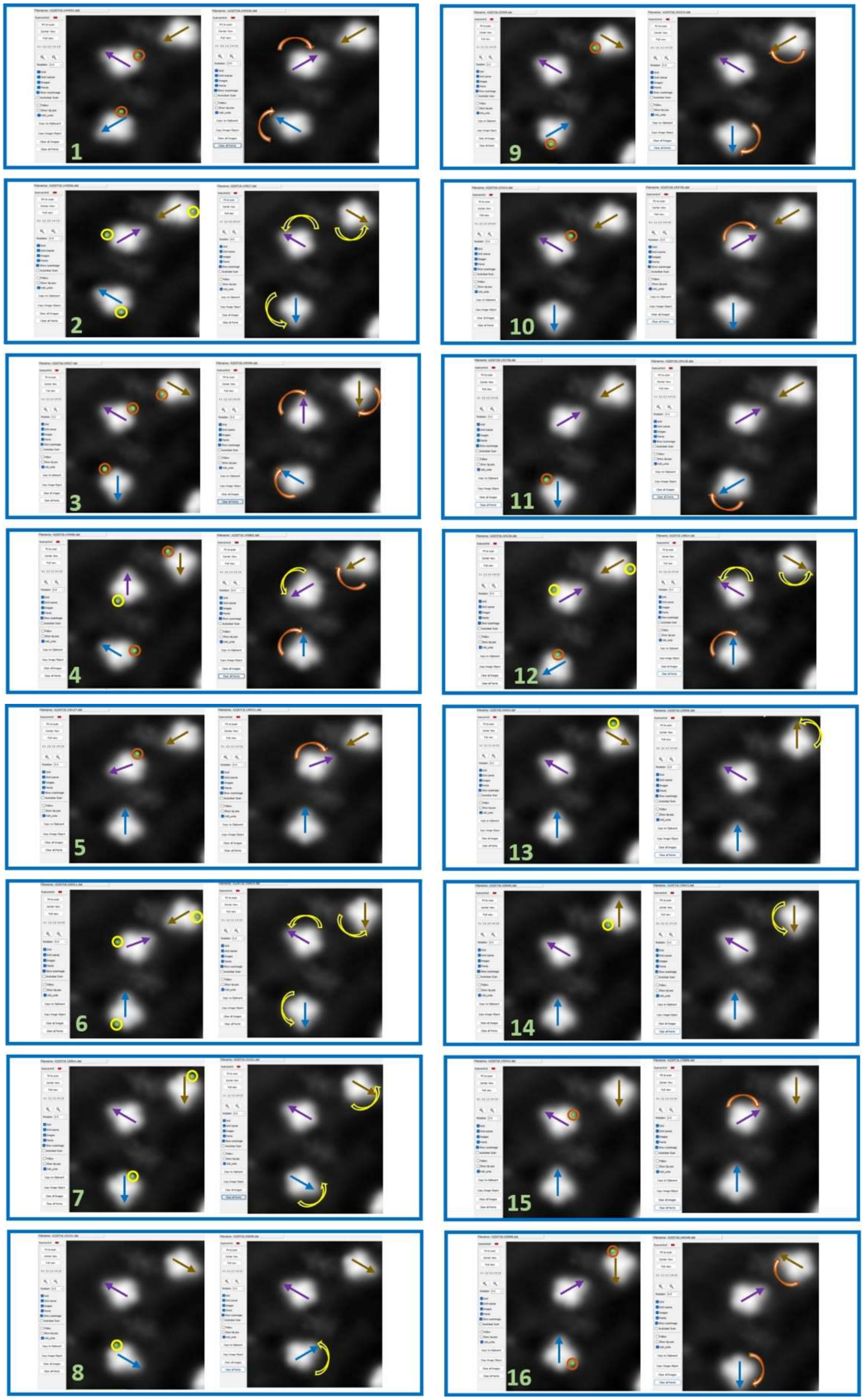

Figure S39. *Rotation sequence 3.*

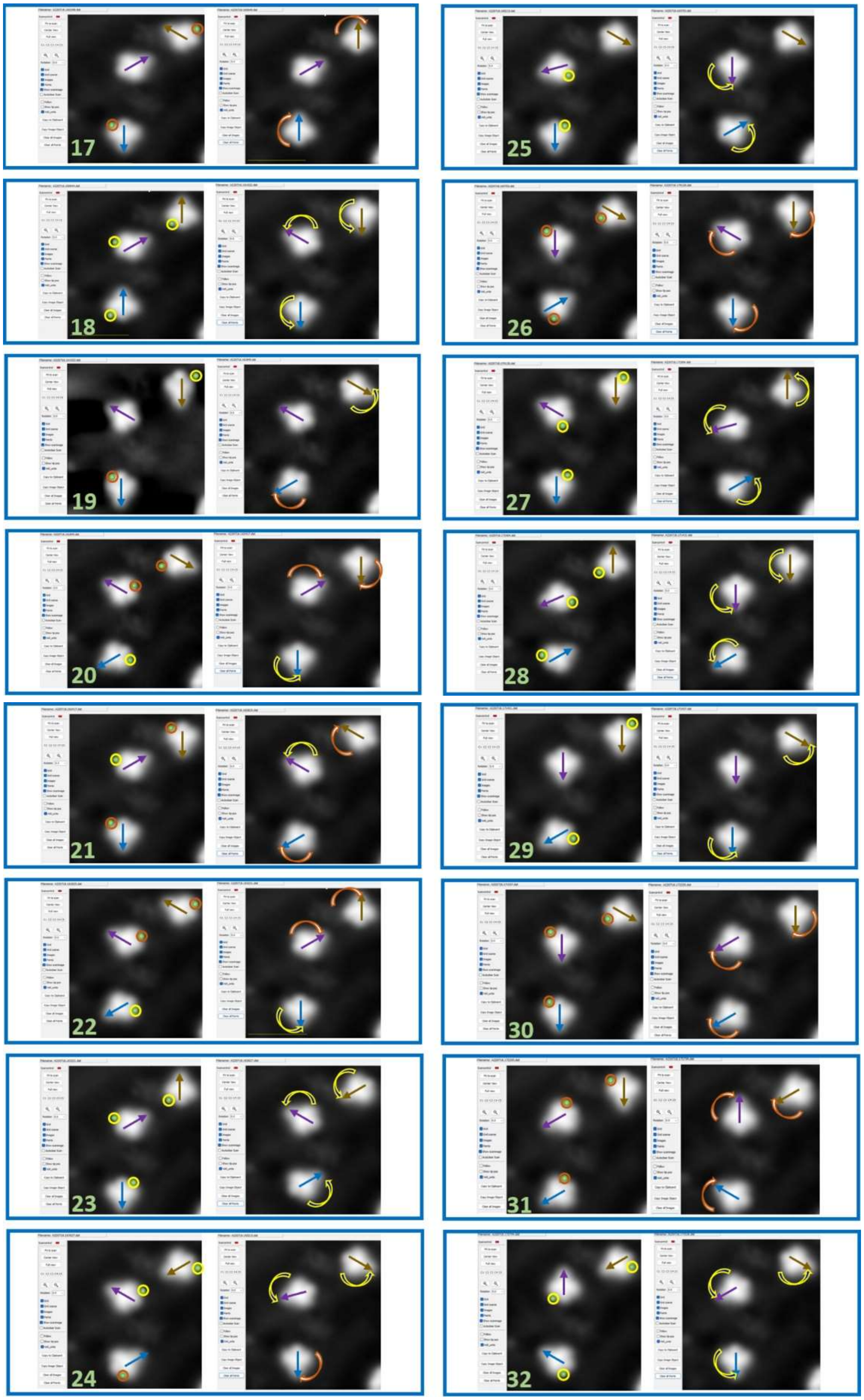

Figure S40. *Rotation sequence 3 continued.*

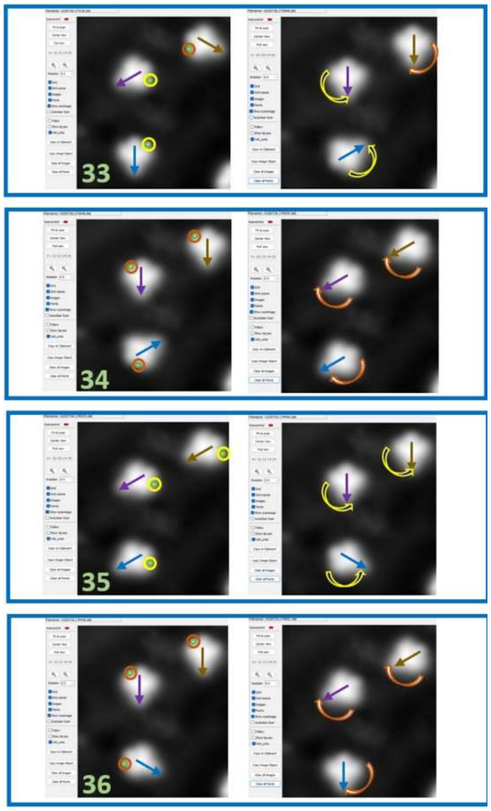

Figure S41. *Rotation sequence 3 continued.*

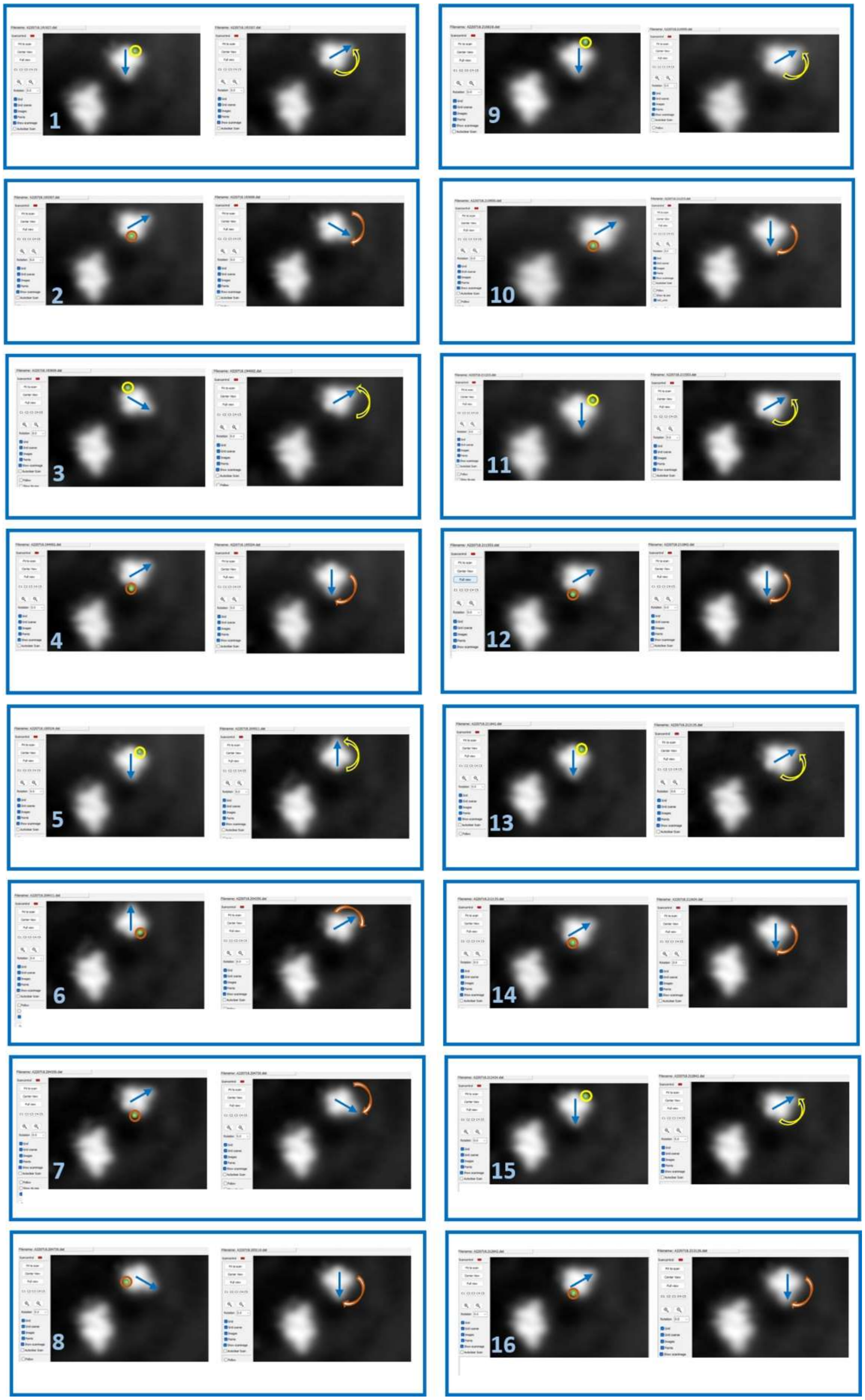

Figure S42. *Rotation sequence 4.*

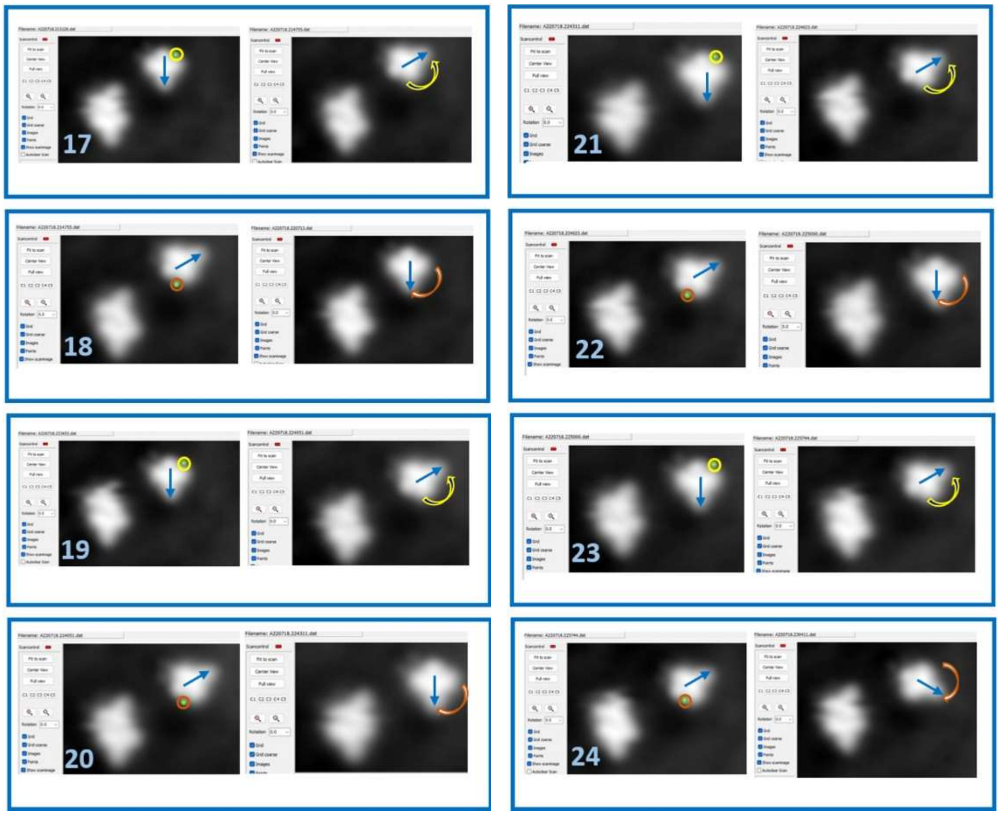

Figure S43. *Rotation sequence 4 continued.*

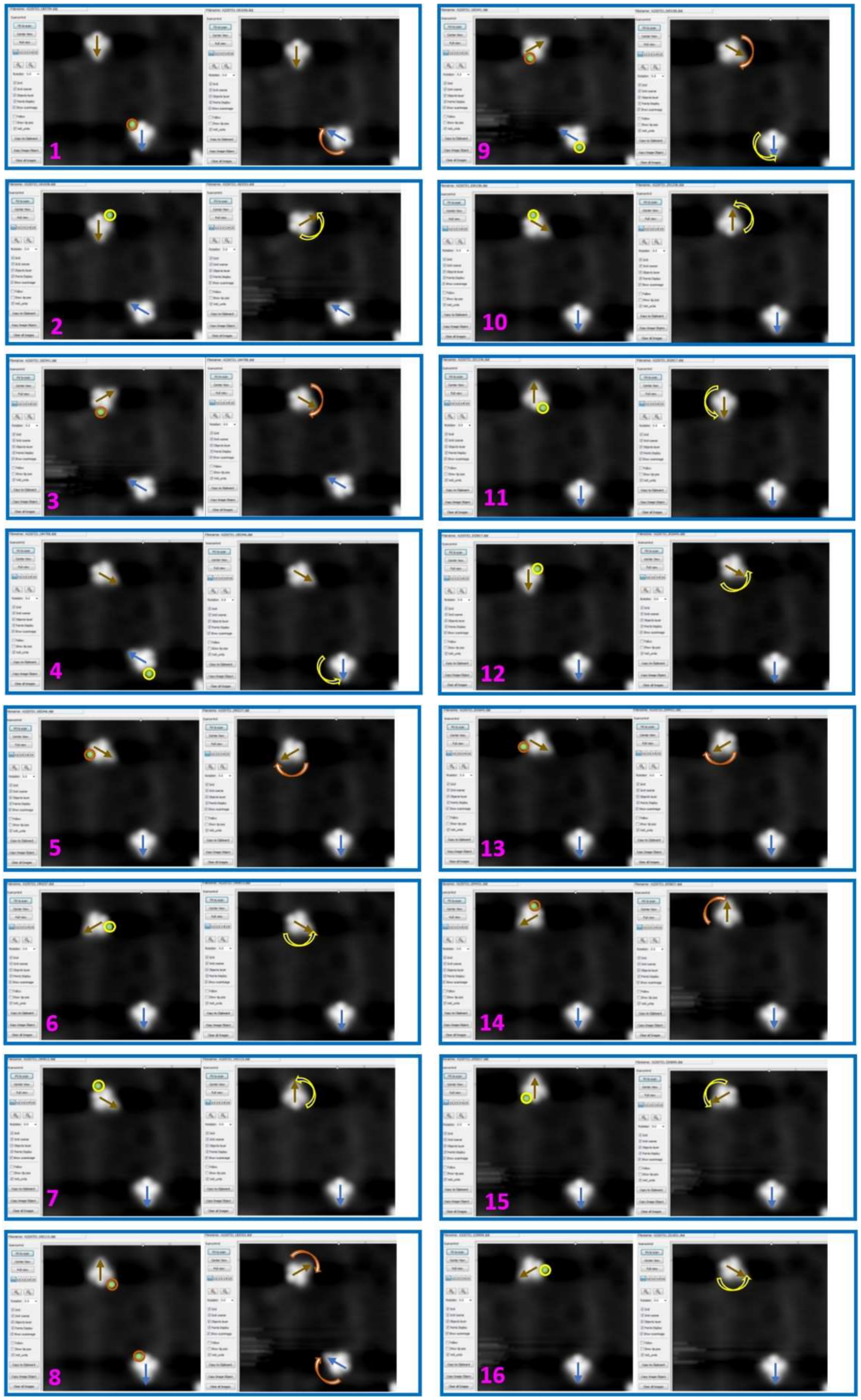

Figure S44. *Rotation sequence 5.*

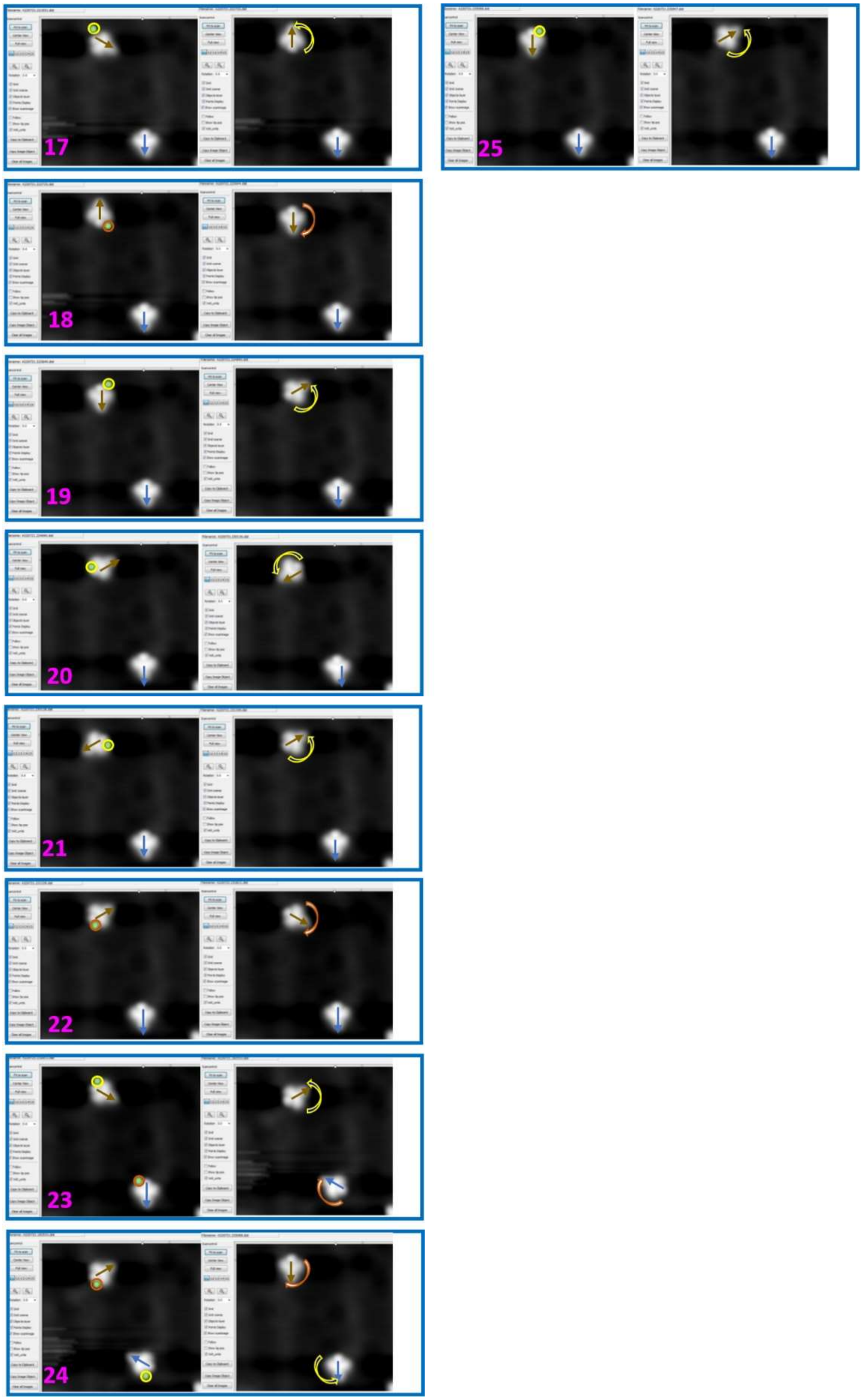

Figure S45. *Rotation sequence 5 continued.*

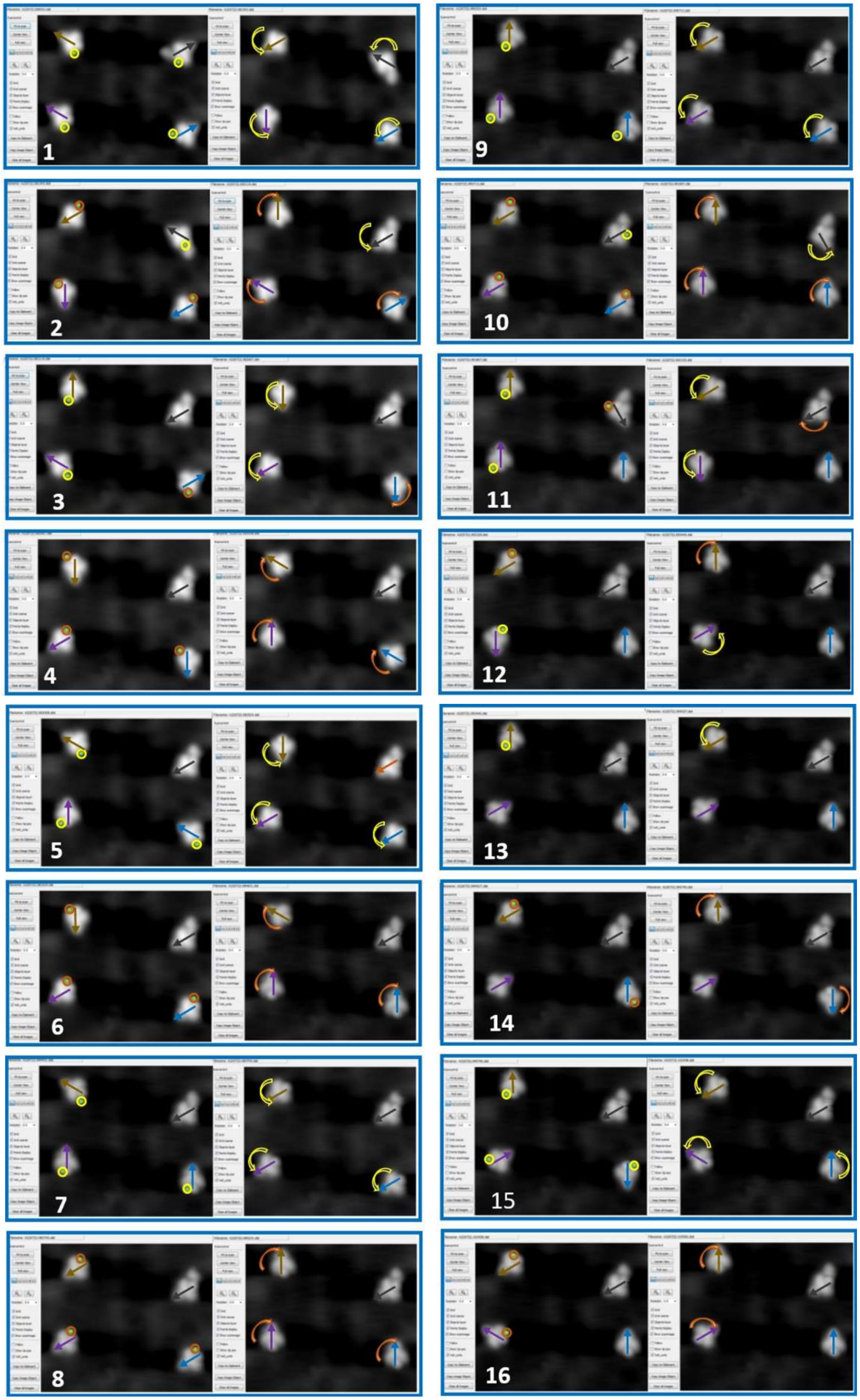

Figure S46. *Rotation sequence 6.*

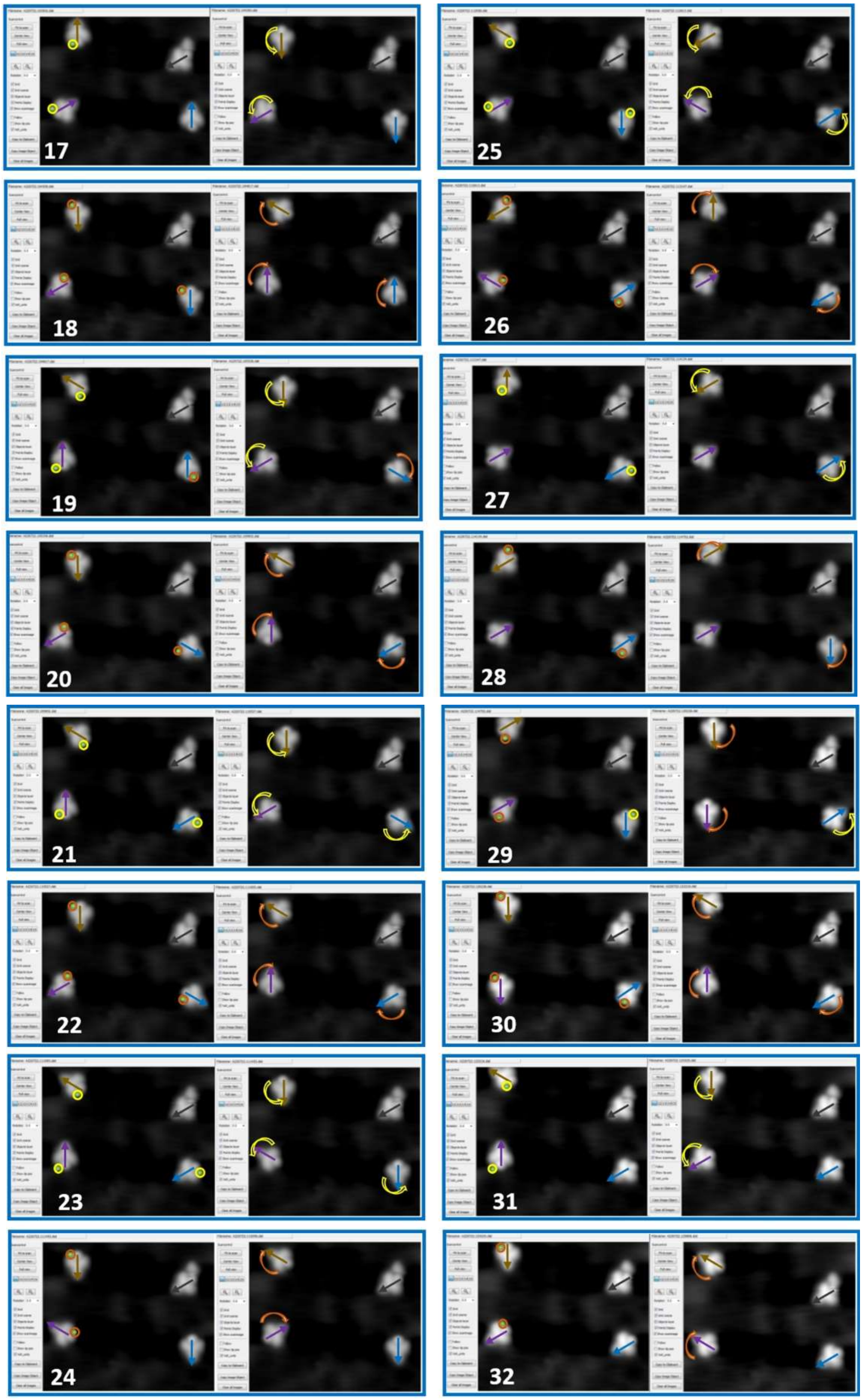

Figure S47. *Rotation sequence 6 continued.*

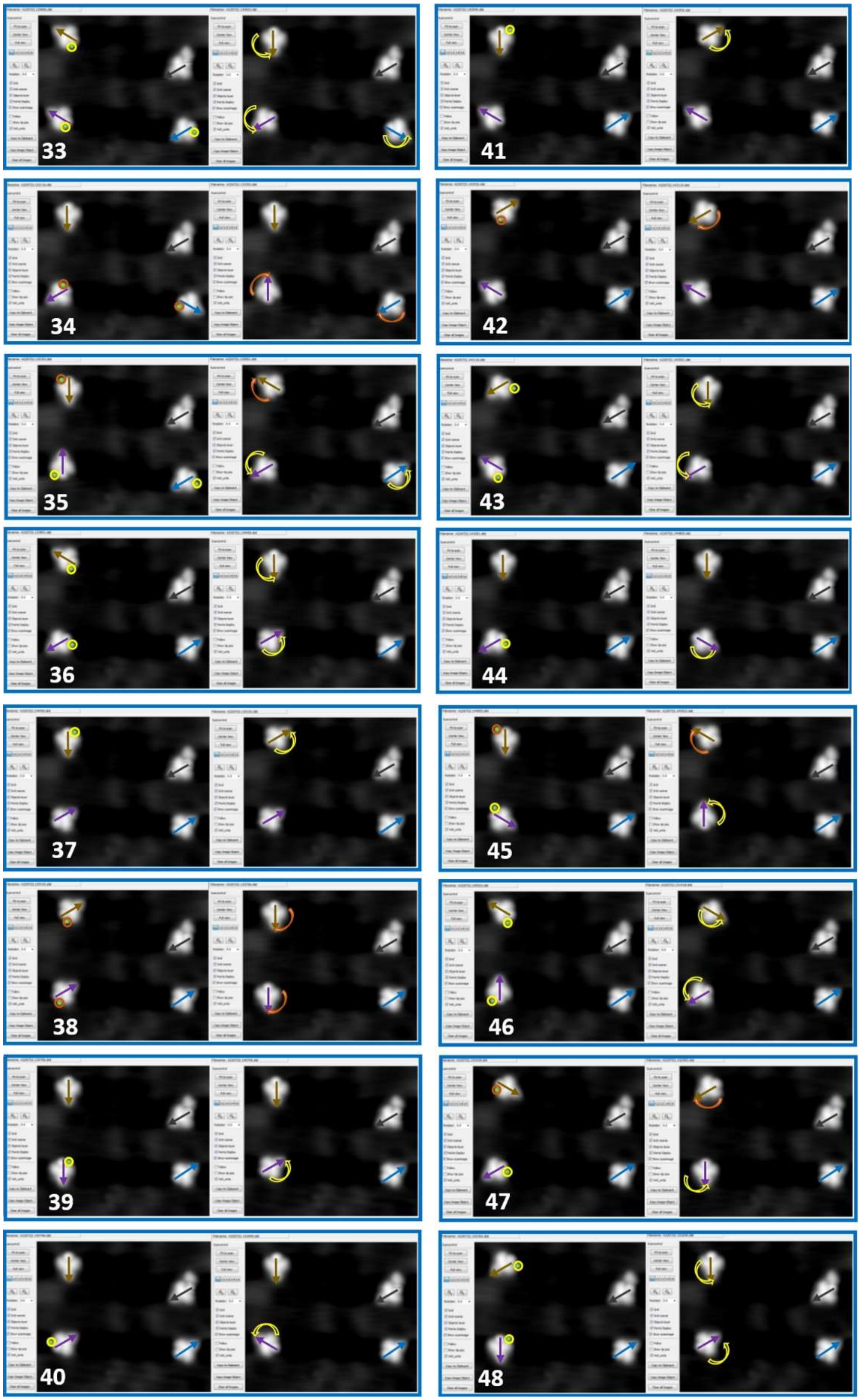

Figure S48. *Rotation sequence 6 continued.*

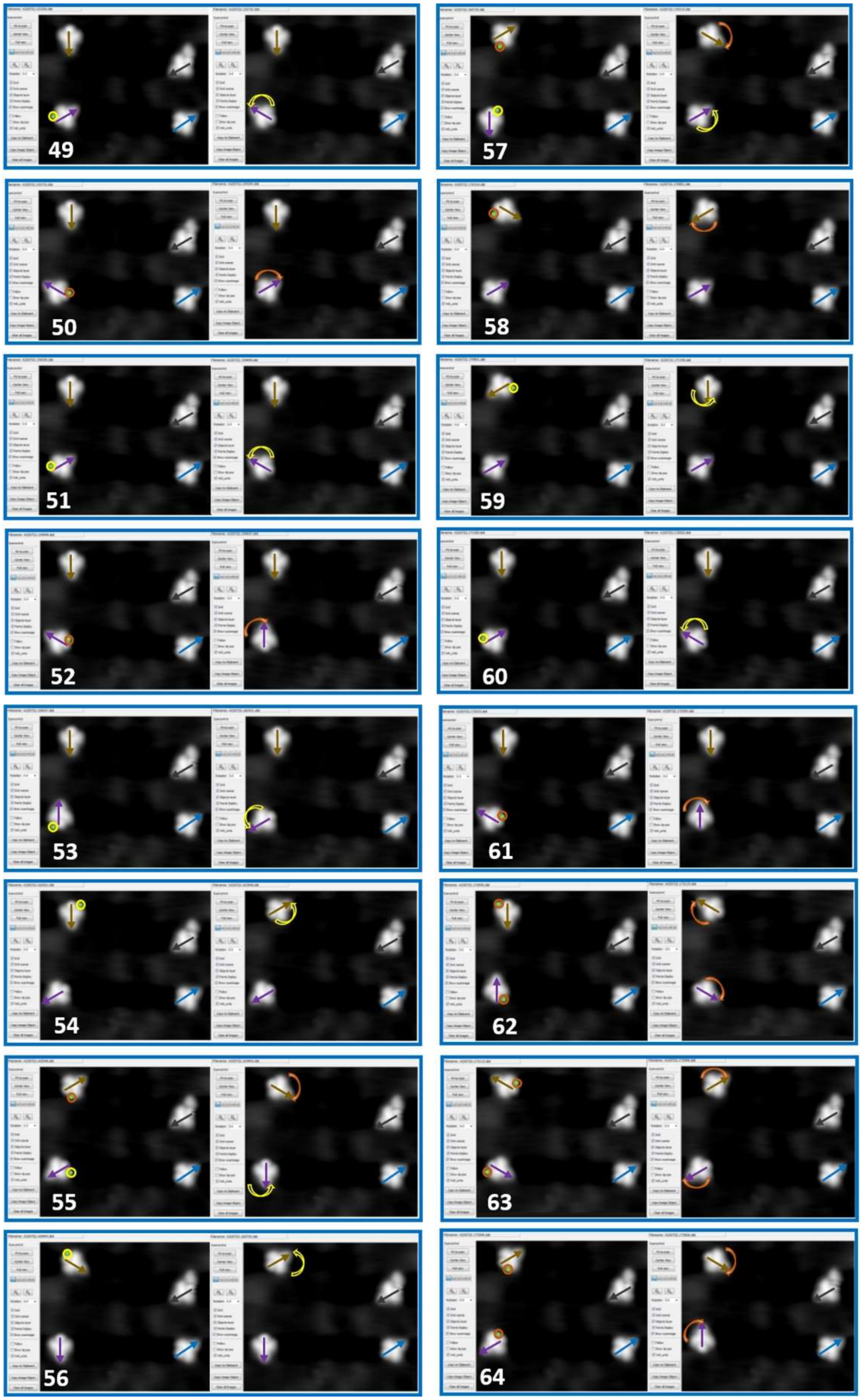

Figure S49. *Rotation sequence 6 continued.*

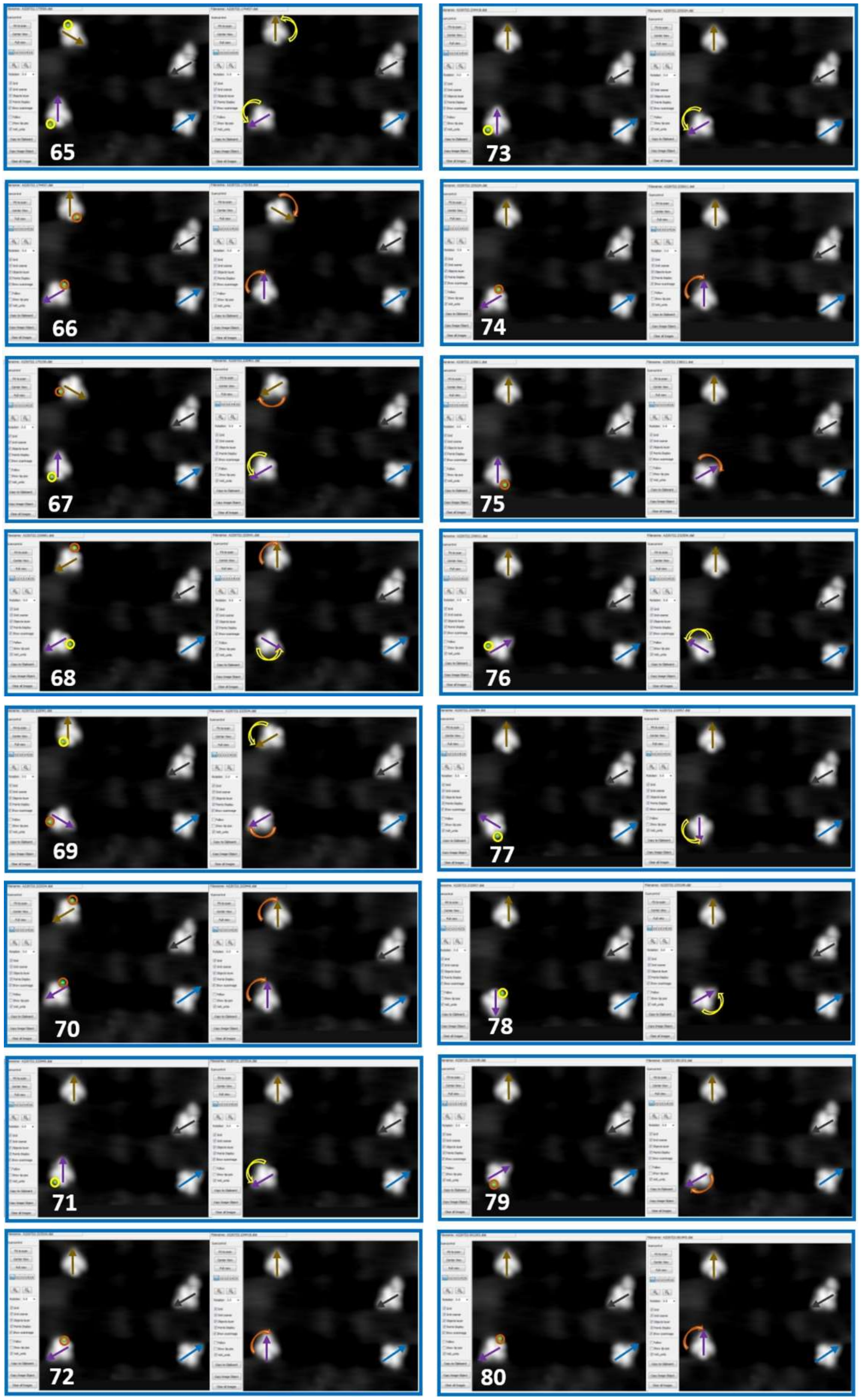

Figure S50. *Rotation sequence 6 continued.*

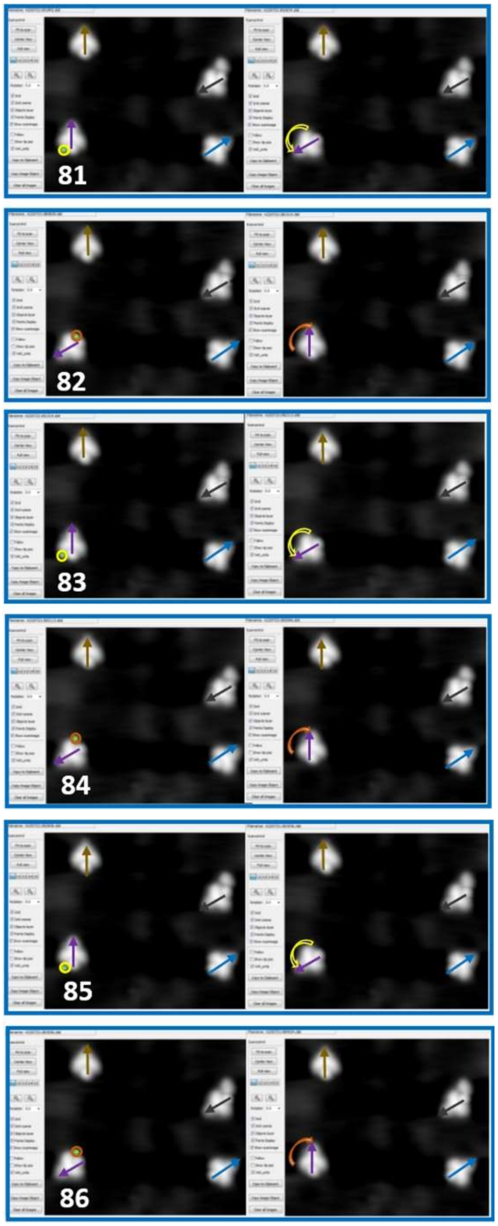

Figure S51. *Rotation sequence 6 continued.*

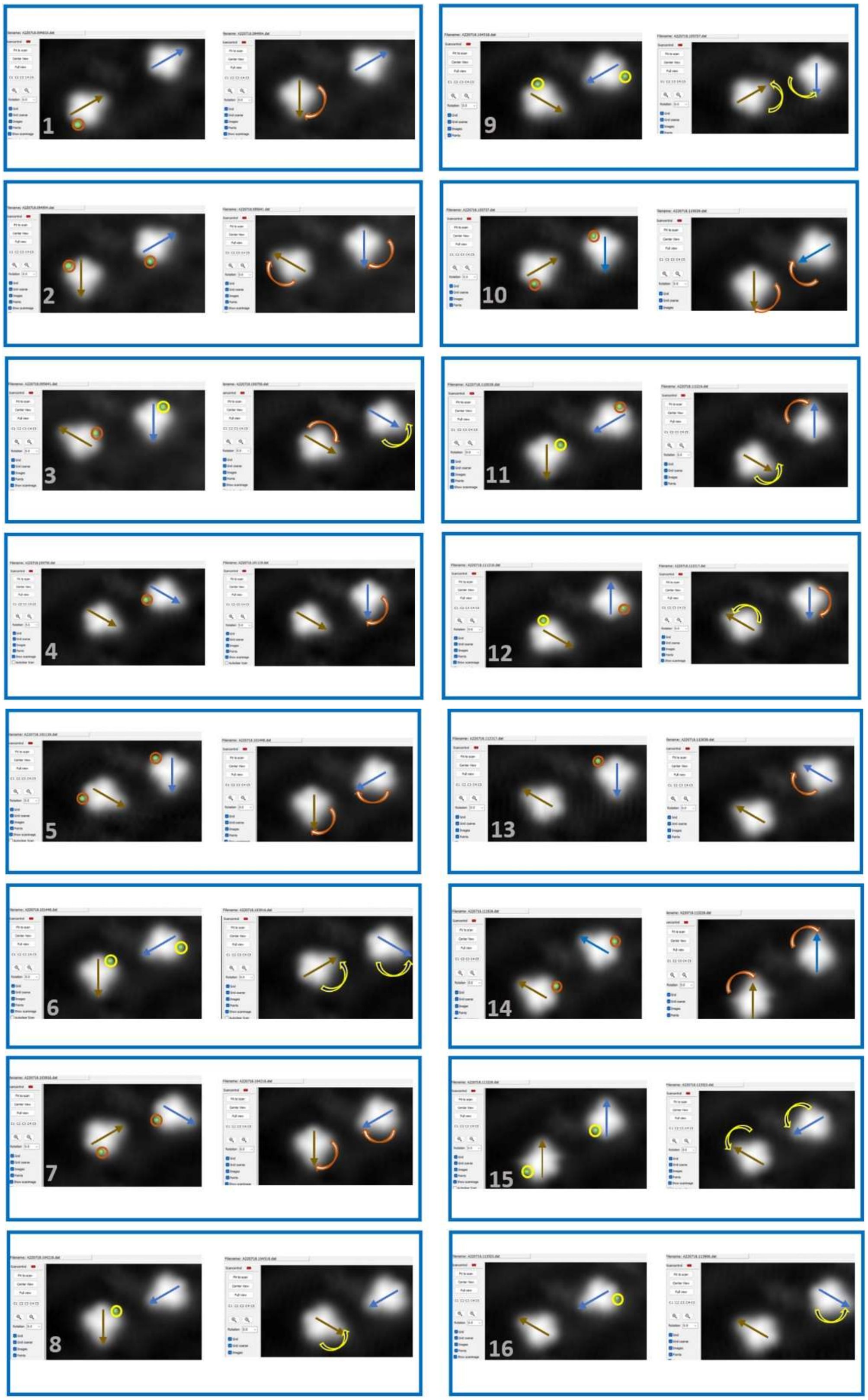

Figure S52. *Rotation sequence 7.*

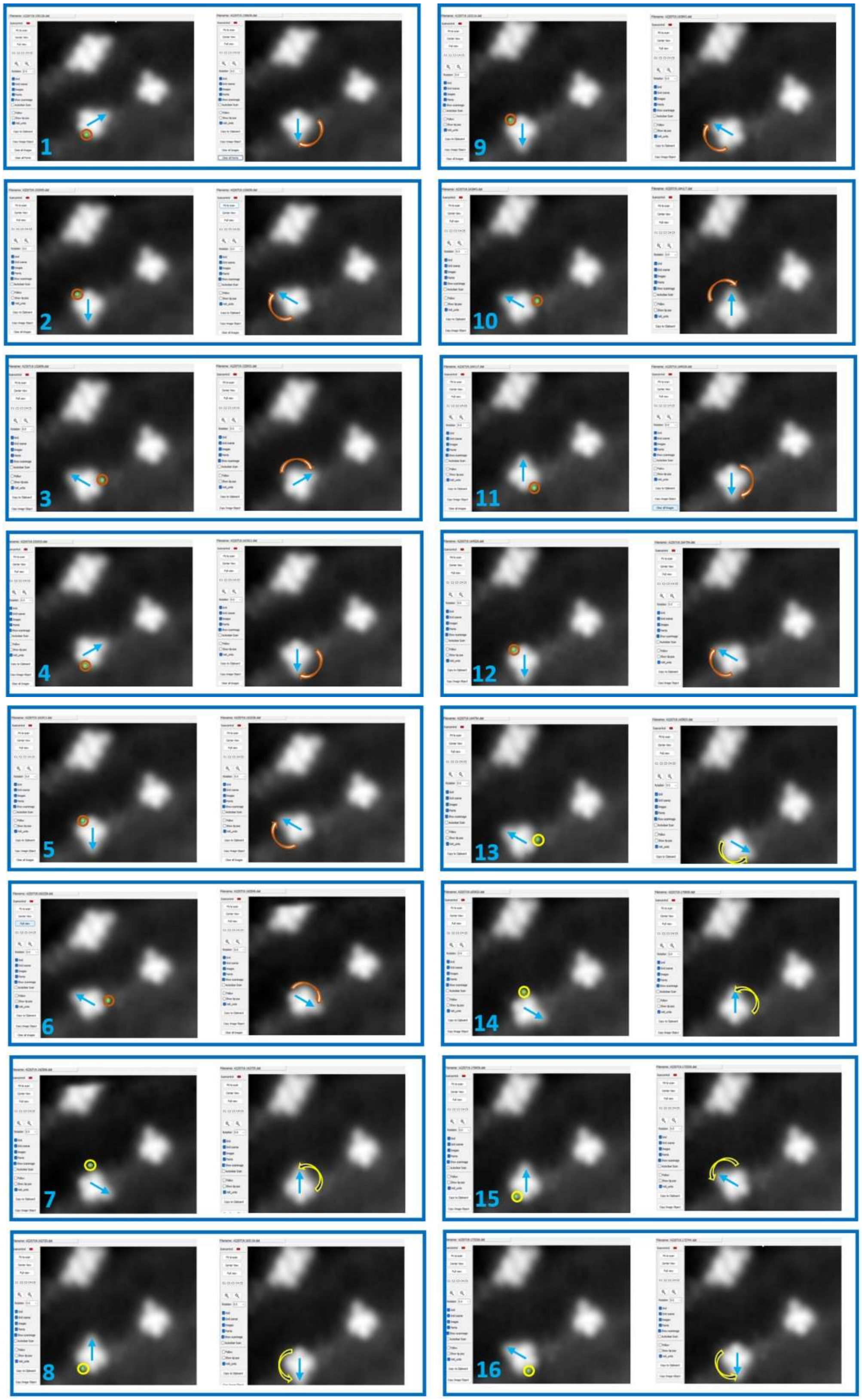

Figure S53. *Rotation sequence 8.*

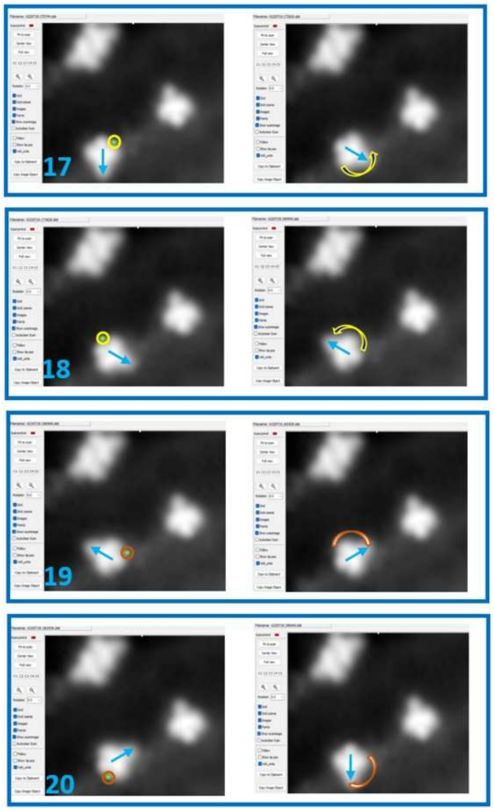

Figure S54. *Rotation sequence 8 – continued.*

Supplementary Note 7. Evidence of Side Counterion Control Over the Complex

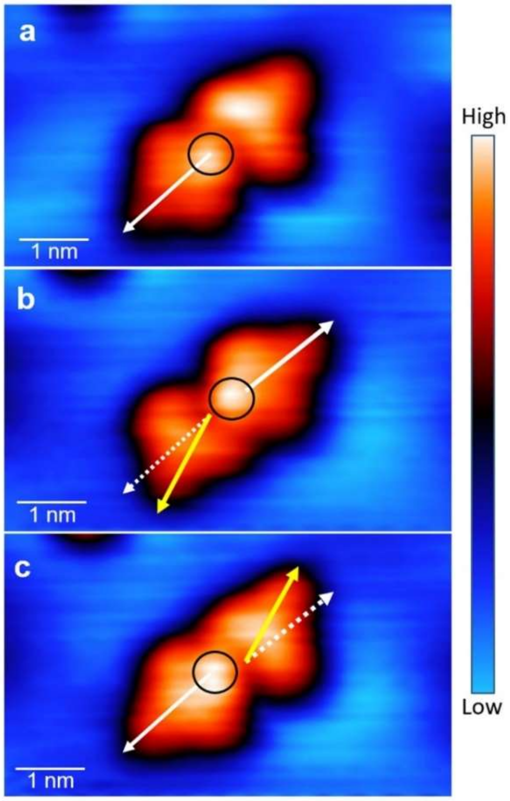

Figure S55. *Control by the counterion.* ***(a)*** *STM image of a pair of* $[Eu(pcam)_3X]^{2+}$ *and* $[Eu(pcam)_3X_2]^{+}$. ***(b)*** *The side counterion from the lower complex is switched to the upper complex.* ***(c)*** *The counterion is switched back to the lower complex. The location of the side counterion is indicated with an oval. The white arrows are along [211] surface directions, the dashed arrows indicate previous orientation and the yellow arrows point the new orientations.* [V_t = -1.0V, I_t = 4 x 10^{-11}A, 5K].

Figure S55a shows an STM image of $[Eu(pcam)_3X]^{2+}$ (upper structure) and $[Eu(pcam)_3X_2]^{+}$ (lower structure) positioned next to each other. During scanning for imaging with a negative bias of -1V, the side counterion of lower complex is switched to the upper complex. Thus, the pair now switches as $[Eu(pcam)_3X]^{2+}$ (lower complex) and $[Eu(pcam)_3X_2]^{+}$ (upper complex) (Fig. S55b). This counterion switching is caused just by the electric field of the scanning STM tip during scanning. After switching, the opposite arm of the upper complex rotates to point towards the exact opposite direction of the first complex with side ion attached (indicated with white arrows in Fig. S55a, and b). After losing the side counterion, the lower complex is also slightly rotated. In the next image (Fig. S55c), the side counterion is switched back to the lower complex. This results in the reorientation of the opposite arm of the complex to its original direction. This sequence of STM images proves that the side counterion dictates the arm directions of the complex.

Supplementary References

[1] Frisch, M. J. et al. *Gaussian 16*.

[2] Kresse, G., & Furthmuller, J. Efficiency of ab-initio total energy calculations for metals and semiconductors using a plane-wave basis set. *Comput. Mat. Sci.* **6**, 15-50 (1996).

[3] Perdew, J.P., Burke, K., & Ernzerhof, M. Generalized gradient approximation made simple. *Phys. Rev. Lett.* **77**, 3865-3868 (1996).

[4] Adamo, C., & Barone, V. Toward reliable density functional methods without adjustable parameters: The PBE0 model. *J. Chem. Phys* **110**, 6158–6170 (1999).

[5] Gulde, R., Pollak, P., & Weigend, F. Error-balanced segmented contracted basis sets of double-ζ to quadruple-ζ valence quality for the lanthanides. *J. Chem. Theory Comput.* **8**, 4062–4068 (2012).

[6] Weigend, & F., Ahlrichs, R. Balanced basis sets of split valence, triple zeta valence and quadruple zeta valence quality for H to Rn: Design and assessment of accuracy. *Phys. Chem. Chem. Phys.* **7**, 3297 (2005).

[7] Frisch, E. rt al. *GaussView, Version 5.0.8.*; Gaussian Inc, Gaussian Inc.

[8] Tang, W., Sanville, E., & Henkelman, G. A grid-based Bader analysis algorithm without lattice bias. *J. Phys.: Condens. Matter* **21**, 084204 (2009).

[9] Sanville, E., Kenny, S.D., Smith, R., & Henkelman, G. An improved grid-based algorithm for Bader charge allocation, *J. Comp. Chem.* **28**, 899-908 (2007).

[10] Henkelman, G., Arnaldsson, A., & Jónsson, H. A fast and robust algorithm for Bader decomposition of charge density, *Comput. Mater. Sci.* **36**, 354-360 (2006).

[11] Yu, M., & Trinkle, D. R. Accurate and efficient algorithm for Bader charge integration, *J. Chem. Phys.* **134**, 064111 (2011).
