# Supplementary material for: Atomically precise control of rotational dynamics in charged rare-earth complexes on a metal surface
Source: Nat Commun. 2022 Oct 22;13:6305. doi: 10.1038/s41467-022-33897-3 (PMC9588029; doi:10.1038/s41467-022-33897-3)
Supplement: Supplementary file 3 — Description of Additional Supplementary Files [file 41467_2022_33897_MOESM3_ESM.pdf]

Supplementary Movie 1: Orbital Mapping of $[Eu(pcam)_3X]^{2+}$ and $[Eu(pcam)_3]^{3+}$. This movie reveals different energetic positions, and the shapes of unoccupied orbitals of $[Eu(pcam)_3X]^{2+}$ and $[Eu(pcam)_3]^{3+}$. It is created from 8000 dI/dV spectroscopic maps acquired over a pair of $[Eu(pcam)_3X]^{2+}$ - $[Eu(pcam)_3]^{3+}$ complexes at ±2000 mV range with 1 mV interval between the consecutive frames.

Supplementary Movie 2: Clockwise Rotation. This movie shows controlled clockwise rotation of an $[Eu(pcam)_3X_2]^{+}$ complex on Au(111) surface when a negative electric field is applied from the STM tip.

Supplementary Movie 3: Anticlockwise Rotation. This movie shows controlled anticlockwise rotation of an $[Eu(pcam)_3X_2]^{+}$ complex on Au(111) surface when a negative electric field is applied from the STM tip.
